# Mapping Machine Learning–Driven Cybersecurity Solutions in Health Care: Scoping Literature Review

**DOI:** 10.2196/93950

**Published:** 2026-07-27

**Authors:** Kunal Rajput, Sharukh Zuberi, Mireille Elhajj, Washington Ochieng, Ara Darzi, Saira Ghafur

**Affiliations:** 1 Department of Surgery and Cancer, Faculty of Medicine, Imperial College London, Faculty Building, South Kensington Campus, London, England, SW7 2AZ, United Kingdom, 44 02075895111; 2 Department of Surgery, The Royal Free Hospital, London, England, United Kingdom; 3 Department of Civil and Environmental Engineering, Faculty of Engineering, Imperial College London, London, England, United Kingdom; 4 Astra-Terra Limited, London, England, United Kingdom; 5 Centre for Active Resilience and Security, Imperial College London, London, England, United Kingdom

**Keywords:** machine learning, artificial intelligence, cybersecurity, healthcare systems, intrusion detection, data protection, digital health

## Abstract

**Background:**

Health care systems face escalating cyberattacks, including the UK Synnovis ransomware attack, which halted pathology services for 14 weeks; the Ascension Health breach affecting 5.6 million patients; and the Change Healthcare breach costing US $2.5 billion. Conventional cybersecurity measures in health care remain reactive and inadequate against evolving threats. Machine learning (ML) offers adaptive, predictive, real-time cyber defense; yet, there is limited clarity on how ML tools are applied across cybersecurity domains, their real-world effectiveness, and where gaps remain.

**Objective:**

This study aims to map ML applications in health care cybersecurity against the National Institute of Standards and Technology Cybersecurity Framework version 2.0, summarize ML performance, and identify research gaps and implementation considerations.

**Methods:**

A systematic search of Ovid MEDLINE, Embase, and Scopus was conducted on July 30, 2025, for studies between 2019 and 2025. Eligible studies applied ML-based approaches to organizational-level cybersecurity in health care settings, with outcomes related to data privacy or cybersecurity strengthening. Studies on smart devices, blockchain, or those lacking empirical data were excluded. Title and abstract and full-text screening were conducted independently by 2 (KR and SZ) reviewers following the Arksey and O’Malley framework and PRISMA-ScR (Preferred Reporting Items for Systematic Reviews and Meta-Analyses extension for Scoping Reviews) guidelines, with discrepancies resolved by consensus. Data were synthesized narratively and mapped against the 6 National Institute of Standards and Technology Cybersecurity Framework version 2.0 functions (Identify, Protect, Detect, Respond, Recover, and Govern).

**Results:**

From 10,348 articles identified, 45 studies across 18 countries were included, applying 80 ML models. Most studies addressed “Protect” (n=22, 48.9%), encompassing federated learning, homomorphic encryption, and deidentification pipelines. “Detect” (n=13, 28.9%) covered intrusion detection and anomaly-based threat detection. “Identify” (n=8, 17.8%) addressed risk assessment and vulnerability prediction. Only 2 studies addressed “Respond,” and none addressed “Recover” or “Govern.” Classical ML, deep learning, and natural language processing predominated, with intrusion detection being the most common application (n=29). Despite strong controlled performance, only one study demonstrated real-world deployment; most rely on synthetic or outdated benchmark datasets and inconsistent reporting metrics.

**Conclusions:**

To our knowledge, this is the first scoping review to map ML-driven cybersecurity solutions against all six National Institute of Standards and Technology Cybersecurity Framework version 2.0 functions, offering a structured, policy-relevant evidence base. ML applications remain concentrated in preincident functions, with gaps in response, recovery, and governance. This highlights systematic blind spots and priorities for researchers and implementers. The evidence supports a phased implementation approach: beginning with detection systems, where evidence is most established and integration is most feasible, progressing to privacy-preserving architectures, for which the literature currently offers no guidance. Implementation requires sustained investment in infrastructure, representative datasets, explainable AI, and real-world validation. Limitations include language bias, methodological heterogeneity, and exclusion of medical devices and proprietary solutions.

## Introduction

### Background

The digitization of health care through networked health information systems, electronic health record (EHR) infrastructure, cloud storage, and telemedicine has transformed care delivery but simultaneously expanded the attack surface, making the sector increasingly vulnerable to sophisticated cyberattacks [1]. Recent high-profile incidents illustrate the scale of disruption. The Synnovis ransomware attack in Southeast London disrupted pathology services across 5 major hospitals and 194 primary care practices, resulting in a 14-wk suspension of blood product processing and delayed clinical diagnostics [2]. The Ascension Health ransomware attack affected 5.6 million patients’ records and led to widespread disruption of clinical services, including delayed treatments and appointments [3]. Change Healthcare, a subsidiary of UnitedHealth Group, incurred economic losses estimated at nearly US $2.5 billion due to billing and prescription shutdowns, highlighting the direct financial impact of cyber incidents [4]. These incidents demonstrate that cyberattacks in health care can have critical impacts across multiple layers, including patient care, financial stability, and operational continuity.

Conventional cybersecurity tools, such as firewalls, antivirus software, intrusion detection systems, and access controls, rely on static rules and known threat signatures to identify and block attacks [5]. Over time, as health care systems have become increasingly digitalized, these tools have evolved to include more sophisticated features, such as real-time monitoring, automated patch management, and basic anomaly detection. However, their fundamentally reactive design limits their ability to address novel or rapidly evolving cyber threats. In contrast, machine learning (ML)-based systems have shown potential to offer a more adaptive, predictive, and real-time approach to cyber defense. These models can detect anomalies without relying on predefined attack signatures and can automate responses, providing scalable and timely defense. This capability is especially important in health care, where even brief outages can threaten patient safety [6].

AI represents a double-edged sword in cybersecurity. On one edge, malicious actors are increasingly using AI to orchestrate sophisticated cyberattacks capable of propagating quickly across interconnected health care systems and disrupting critical services [7,8]. Emerging threat intelligence reports suggest that AI tools have been used as autonomous penetration testing orchestrators, achieving high levels of autonomous execution across the cyberattack lifecycle [8]. Almost three-quarters of phishing attacks now leverage AI technology, with some estimates suggesting that the majority of phishing emails now incorporate AI in some form, whether for text generation, personalization, or obfuscation [9]. Conversely, AI also offers powerful defensive capabilities, enabling health care systems to adopt equally advanced AI-driven defense mechanisms that can detect anomalies in real time and automate responses to emerging threats [10,11]. This technological arms race, where both attackers and defenders leverage AI, underscores the critical need for health care organizations to not only implement AI-based security solutions but also establish robust governance frameworks and regulatory oversight to ensure these systems are deployed responsibly, monitored continuously, and protected against adversarial exploitation.

To date, AI adoption in health care has been predominantly concentrated on clinical domains such as medical imaging interpretation, pathology, and predictive analytics, with a strong emphasis on improving diagnostic accuracy and workflow efficiency [12]. ML adoption in health care cybersecurity remains limited due to the evolving complexity of cyber threats, fragmented regulation, and reliance on human expertise for real-time decisions [13]. Unlike diagnostics, where clear regulatory pathways exist, cybersecurity lacks a unified framework for ML integration. This review focuses specifically on ML, a subset of AI in which systems learn patterns from data, encompassing classical algorithms, deep learning (DL), and natural language processing (NLP). While Internet of Medical Things and smart device security represent a related concern, this review focuses on organizational-level health care systems, where ML-based cybersecurity solutions are most developed.

The UK’s Cyber Security Strategy for Health and Adult Social Care (2023‐2030) highlights the use of AI for intelligent threat detection and system resilience [14]. Similarly, the US National Cybersecurity Strategy (2023) advocates for AI-driven tools to enable real-time anomaly detection and adaptive defense. These policies reflect a growing shift toward proactive, AI-enabled cybersecurity strategies [15].

The National Institute of Standards and Technology Cybersecurity Framework (NIST-CSF) 2.0, released in 2024, reinforces this shift toward proactive and adaptive cyber defense [16]. The six pillars “Identify,” “Protect,” “Detect,” “Respond,” “Recover,” and “Govern” together emphasize strategic oversight, continuous improvement, and integration of emerging technologies such as ML. For health care systems, NIST-CSF offers a flexible structure to align cybersecurity efforts with operational demands, providing a domain framework for the adoption of intelligent, AI-enabled tools for real-time detection and response.

Several reviews have examined the application of AI and ML to cybersecurity more broadly. Wiafe et al [17] conducted a systematic mapping of AI for cybersecurity across general computing environments, cataloging techniques across domains including intrusion detection, malware analysis, and network security. Ali et al [18] provided a systematic review of AI and ML techniques for cybersecurity, and a subsequent review by the same group focused specifically on DL methods for malware and intrusion detection [19]. Dhanushkodi and Thejas [20] examined AI-enabled threat detection across general security contexts, highlighting advances in anomaly-based and signature-free detection. While these reviews provide valuable insights into the broader AI-cybersecurity landscape, they are not specific to health care and do not account for the sector’s unique constraints such as the sensitivity of patient data, the criticality of service continuity, legacy infrastructure, and the complex regulatory environment governing health systems. Furthermore, existing reviews tend to focus on particular technical domains, such as intrusion detection or malware classification, without offering a comprehensive mapping of how the full breadth of ML tools aligns with an established cybersecurity governance framework applicable to health care [21,22].

No prior scoping review has systematically mapped ML applications in health care cybersecurity against the NIST CSF 2.0, which captures all phases of the cyber resilience lifecycle. This review addresses that gap by providing the first governance-aligned, health care–specific synthesis of ML cybersecurity evidence organized against NIST CSF 2.0, with the explicit aim of informing organizational implementation priorities and identifying where the evidence base is critically absent.

### Objectives

This scoping review aims to systematically map ML applications to health care cybersecurity tools. The specific objectives are to:

1. Identify and categorize ML techniques developed in health care cybersecurity
2. Map ML applications across the six NIST CSF 2.0 functions (Identify, Protect, Detect, Respond, Recover, and Govern)
3. Characterize the health care environments and systems addressed by these applications
4. Analyze the methodological approaches to cyber resilience and the geographic distribution of research
5. Identify implementation considerations and highlight research gaps to inform future ML cybersecurity development in health care settings.

## Methods

The structure of this scoping review was guided by the PRISMA-ScR (Preferred Reporting Items for Systematic Reviews and Meta-Analyses extension for Scoping Reviews) checklist (Checklist 1). In addition, search reporting adhered to the PRISMA-S (Preferred Reporting Items for Systematic Reviews and Meta-Analyses literature search extension) guidelines [23]. A completed PRISMA-S checklist is provided in Checklist 2. The review was registered with Open Science Framework (OSF Registries 4cjmu).

Studies were selected based on the Population, Intervention, Comparison, Outcome, Study design criteria. Eligible studies were those involving organization-level cybersecurity in a health care setting, including hospitals, clinics, and health networks, where an ML-based approach was applied to enhance cyber resilience through predictive analytics, anomaly detection, and automated response. Outcomes of interest included assessments of data privacy, protection of health care data, or strengthening of cybersecurity practices. Studies primarily focused on smart or wireless health care devices or blockchain-based cybersecurity were excluded, as were nonpeer-reviewed sources including gray literature, conference abstracts, books, editorials, commentaries, case reports**,** review articles, and theoretical or technical concept papers without empirical evaluation.

A total of 3 electronic databases were searched: Ovid MEDLINE, Embase Classic+ Embase, and Scopus on July 30, 2025. MEDLINE and Embase Classic+ Embase were searched simultaneously via the Ovid platform as a multidatabase search; Scopus was searched separately via its native interface. Ovid MEDLINE and Embase provide comprehensive coverage of the peer-reviewed health care and biomedical literature, while Scopus offers broad multidisciplinary indexing encompassing computer science, engineering, and cybersecurity.

The search strategy was developed de novo in consultation with an expert librarian at Imperial College London and combined both medical subject headings terms for MEDLINE- and Emtree-controlled vocabulary terms for Embase with free-text terms. Boolean operators (AND, OR) were used to combine concept blocks; truncation using the asterisk wildcard and adjacency operators (adj1, adj2) were applied. Keywords included variations of the following: “cybersecurity,” “cyber resilience,” “healthcare,” “health system,” “artificial intelligence,” “machine learning,” “data privacy,” “vulnerability,” “threat,” and “risk.” Searches were limited to English language and human studies between 2019 and 2025. Additional studies were identified through reference list screening from included articles. No websites, tables of contents, or print sources were purposefully browsed, and no authors, experts, or manufacturers were contacted to identify additional studies. Gray literature, study registries, and other sources were not searched, and formal peer review of the search strategy was not conducted. Following reviewer and editor feedback, the search strategy was revised and rerun during the revision process; however, the search end date of July 30, 2025, was retained as the fixed time horizon for the review. No email alerts were used. Full search strategies for all 3 databases are available in Multimedia Appendix 1.

Study selection was conducted in two phases using the Covidence platform (developed by Veritas Health Innovation). A total of two independent reviewers (KR and SZ) conducted titles or abstracts screening and full-text screening. Any discrepancies were resolved through discussion. In keeping with scoping review methodology, no formal critical appraisal or risk of bias assessment was conducted. Data extraction elements were defined prospectively and refined following preliminary searches to provide a comprehensive overview of ML applications in health care cybersecurity and to capture the breadth of study designs, health care settings, ML techniques, and NIST framework classifications. A standardized data extraction form was developed in Microsoft Excel and validated by research team members with expertise in health care cybersecurity and AI (SG and WO). Data extraction was completed by one author (KR) and independently checked by a second reviewer (SZ), with discrepancies resolved through discussion. The extracted information included study characteristics (lead author, year of publication, study design, and country of authorship), AI model type, training data source, performance metrics (eg, accuracy, precision, recall, *F*_1_-score, and area under the receiver operating characteristic), and reported ML-based cyber resilience domain, such as anomaly detection, data privacy protection, intrusion detection, threat analysis, and risk assessment. Performance metrics were extracted as reported by study authors; given heterogeneity in metrics used, validation approaches, and dataset types across studies, cross-study comparison of performance figures was not reported.

Studies were systematically categorized and mapped against the NIST CSF 2.0 functions as the organizing structure, classifying evidence across the six core functions: Identify, Protect, Detect, Respond, Recover, and Govern. Classification was completed by one author (KR) and independently checked by a second reviewer (SZ), with discrepancies resolved through discussion. Each study was assigned to a single NIST CSF 2.0 function based on its primary cybersecurity objective. Within each thematic domain, studies were compared by ML technique, health care environment, cybersecurity application, study design, and key findings. Results are presented using a combination of narrative description and visual representations.

## Results

### Study Characteristics

**Figure 1.**
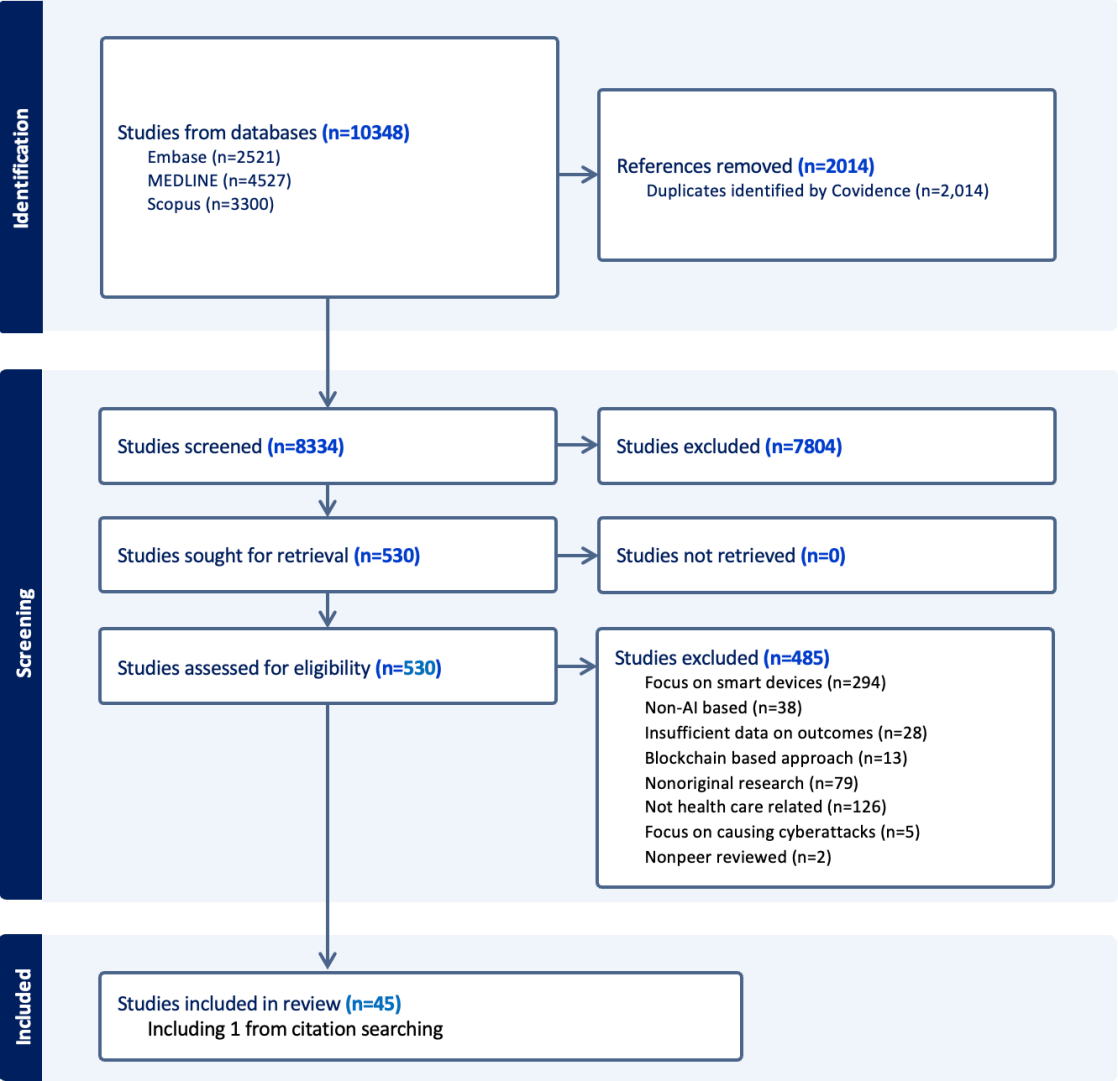
PRISMA-ScR (Preferred Reporting Items for Systematic reviews and Meta-Analyses for Scoping Reviews) flow diagram illustrating the identification, screening, eligibility assessment, and inclusion of peer-reviewed studies examining machine learning applications in health care cybersecurity, drawn from systematic searches of Ovid MEDLINE, Embase, and Scopus conducted on July 30, 2025 (search period: 2019‐2025).

There were 10,348 articles identified from the database search. After removing 2014 duplicates, the remaining records were screened by title and abstract by two independent reviewers (KR and SZ), resulting in 530 articles selected for full-text review. Figure 1 summarizes the PRISMA-ScR flowchart of the study.

Following independent full-text review, 45 [24-68] global studies (including 1 from citation search) were finalized for analysis. These were conducted across 18 countries and applied 80 ML models to diverse datasets in health care cybersecurity. Table 1 summarizes the study characteristics of the included studies. China (7/45, 15.6%) and India (7/45, 15.6%) had the highest representation among included studies. The studies were conducted across diverse health care environments, most commonly health care systems (17/45, 37.8%), followed by electronic health records (7/45, 15.6%), medical data (6/45, 13.3%), health care networks (5/45, 11.1%), and others. ML models were trained on diverse sources, including real-world health care data, cybersecurity or network datasets, and synthetic or benchmark datasets. Included studies varied considerably in dataset size and type, encompassing network traffic samples, patient records, email corpora, NLP token sets, and cyberattack event logs, reflecting the heterogeneous data environments across health care cybersecurity research.

**Table 1.** Characteristics of 45 peer-reviewed studies included in a scoping review of machine learning applications in health care cybersecurity, identified through systematic searches of Ovid MEDLINE, Embase, and Scopus on July 30, 2025 (2019‐2025), categorized by study design, country of first author, and health care environment.

Category	Number of studies (N=45), n (%)	References
Study type		
Quasi-experimental study	41 (91.1)	Silvestri et al [24], Islam et al [25], Li and Wang [26], Yi et al [27], Yusof et al [28], Liu et al [29], Sheu et al [30], Nguyen et al [31], Kumar et al [32], Almousa et al [33], S and S [34], Gao et al [35], Altalla’ et al [36], Mesfer Aldossary [37], Akter et al [38], Jonnagaddala et al [39], Samiayya et al [40], Tabassum et al [41], Hurst et al [42], Saranya et al [43], Öztürk et al [44], Wazid et al [45], Sengan et al [46], Thanh et al [47], Alanazi [48], Ahmed and Shakir [49], Halman and Alenazi [50], Asif et al [51], Qiao et al [52], Fernández Maimó et al [53], Abidi et al [54], Huang et al [58], An et al [59], Bo et al [60], Akter et al [62], Ganguli et al [63], Alzubi et al [64], Cabrero-Holgueras et al [65], Mohammadi et al [66], Goldschmidt et al [67], Gwon et al [68].
Observational study	4 (8.9)	Islam et al [25], Ünözkan et al [55], Dolezel et al [56], Gupta et al [57].
Country of first author		
China	7 (15.6)	Li et al [26], Yi et al [27], Gao et al [35], Qiao et al [52], Huang et al [58], An et al [59], Bo et al [60].
India	7 (15.6)	S and S [34], Samiayya et al [40], Saranya et al [43], Wazid et al [45], Sengan et al [46], Gupta et al [57], Kaur et al [61].
Saudi Arabia	5 (11.1)	Almousa and Uliyan [33], Mesfer Aldossary [37], Alanazi [48], Halman and Alenazi [50], Abidi et al [54].
Australia	5 (11.1)	Liu et al [29], Nguyen et al [31], Akter et al [38], Jonnagaddala et al [39], Akter et al [62].
United Kingdom	3 (6.7)	Islam et al [25], Tabassum et al [41], Hurst et al [42].
Turkey	2 (4.4)	Öztürk et al [44], Wazid et al [45].
United States	2 (4.4)	Dolezel et al [56], Ganguli et al [63].
Jordan	2 (4.4)	[36,64] Altalla’ et al [36], Alzubi et al [64].
Spain	2 (4.4)	Fernández Maimó et al [53], Cabrero-Holgueras [65].
Other countries	10 (22.2)	Silvestri et al [24], Yusof et al [28], Sheu et al [30], Kumar et al [32], Thanh et al [47], Ahmed and Shakir [49], Asif et al [51], Mohammadi et al [66], Goldschmidt et al [67], Gwon et al [68].
Health care environment		
Health care systems	17 (37.8)	Akter et al [38], Jonnagaddala et al, Öztürk et al [44], Wazid et al [45], Sengan et al [46], Alanazi [48], Ahmed and Shakir [49], Halman and Alenazi [50], Asif et al [51], Abidi et al [54], Ünözkan et al [55], Dolezel et al [56], An et al [59], Kaur et al [61], Akter et al [62], Ganguli et al [63], Mohammadi et al [66].
Electronic health records	7 (15.6)	Liu et al [29], Kumar et al [32], Tabassum et al [41], Hurst et al [42], Gupta et al [57], Goldschmidt et al [67], Gwon et al [68]. Jonnagaddala et al [39].
Medical data	6 (13.3)	Sheu et al [30], Nguyen et al [31], Gao et al [35], Altalla’ et al [36], Alzubi et al [64], Cabrero-Holgueras et al [65].
Health care networks	5 (11.1)	Li et al [26], Samiayya et al [40], Saranya et al [43], Thanh et al [47], Qiao et al [52].
Medical images and imaging systems	4 (8.9)	S and S [34], Mesfer Aldossary [37], Huang et al [58], Bo et al [60].
Cyber-physical systems	3 (6.7)	Silvestri et al [24], Yi et al [27], Fernández et al [53].
Health care infrastructure	1 (2.2)	Yusof et al [28].
Employee email	1 (2.2)	Almousa et al [33].
Supply chain	1 (2.2)	Islam et al [25].

**Figure 2.**
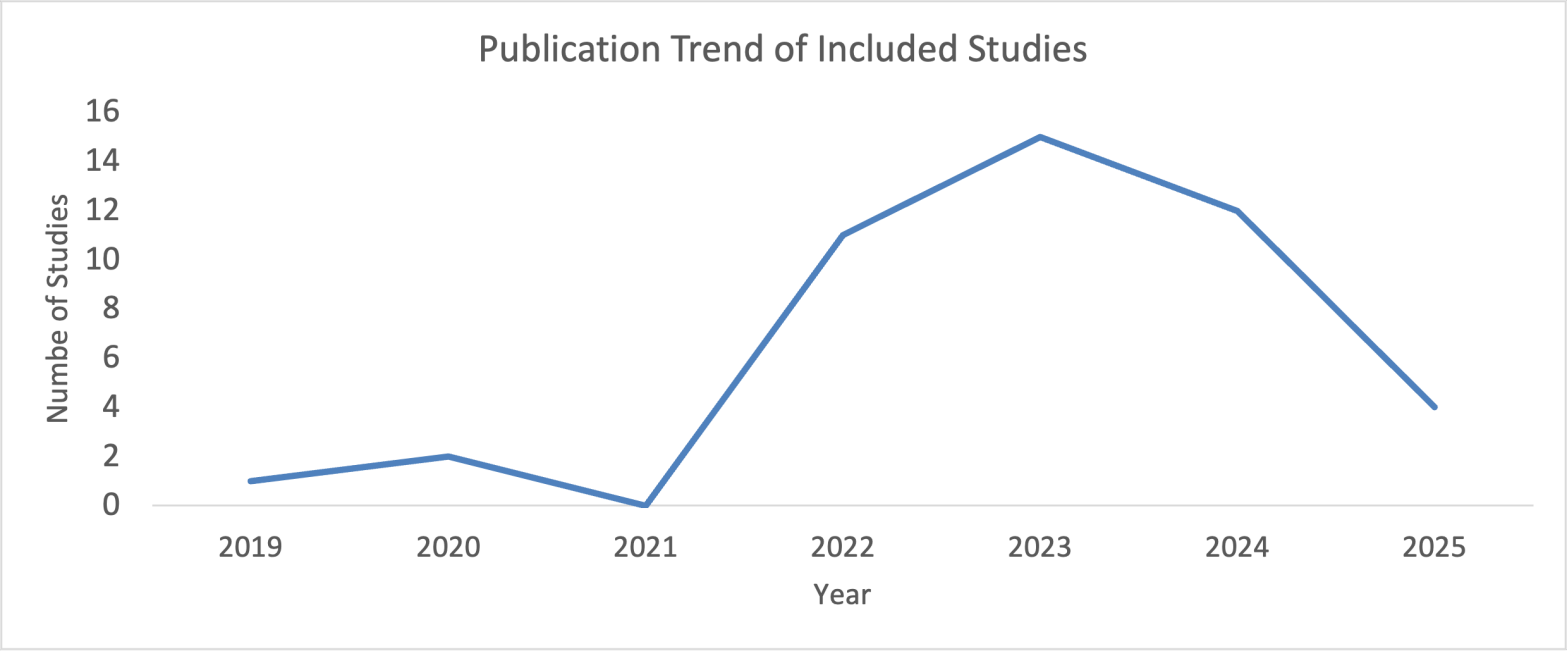
Annual publication trend of 45 peer-reviewed studies examining machine learning applications in health care cybersecurity, identified through systematic searches of Ovid MEDLINE, Embase, and Scopus (search period: 2019‐2025; search date: July 30, 2025), illustrating year-on-year growth in research output from 2019 to 2025.

Figure 2 presents the annual publication volumes. Notably, between 2019 and 2021, the number of publications examining ML and cybersecurity in health care remained consistently low. However, 2022 marked a turning point, with a sharp increase in publications. This upward trend persisted through 2024.

**Figure 3.**
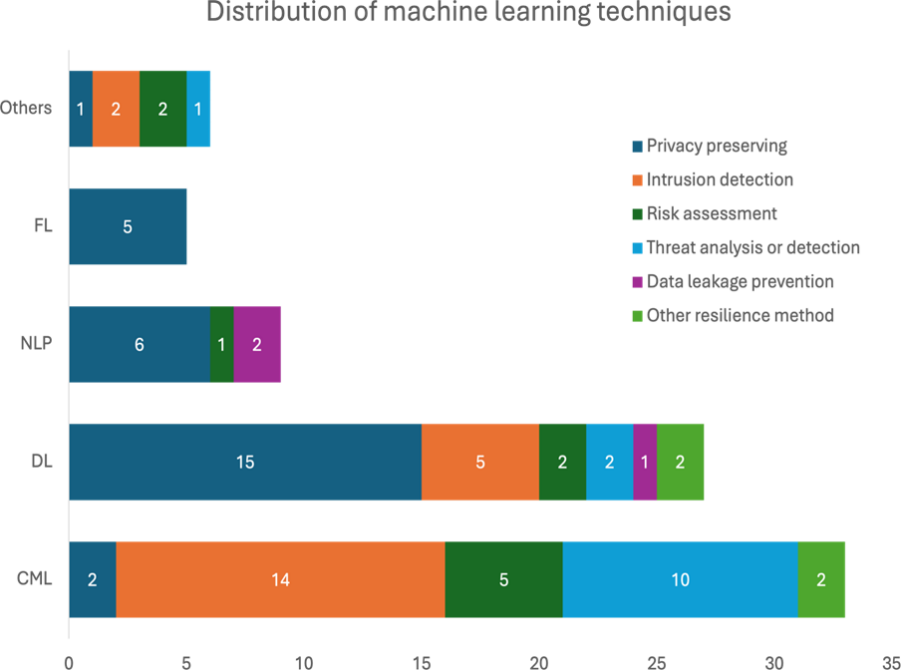
Distribution of machine learning technique categories (classical machine learning, deep learning, natural language processing, and federated learning) across cybersecurity application domains (privacy-preserving, intrusion detection, risk assessment, threat analysis, and data leakage prevention) in 45 peer-reviewed studies of health care cybersecurity (2019‐2025). Multiple ML models were applied in most studies; therefore, the total number of ML techniques (n=80) exceeds the number of reviewed papers (n=45). CML: classic machine learning; DL: deep learning; FL: federated learning; NLP: natural language processing.

The distribution of ML techniques across cybersecurity applications is summarized in Figure 3. Classical ML (n=33) and DL (n=27) demonstrated the widest application breadth, with both predominantly used for privacy prevention (n=14) and intrusion detection (n=15). Overall, intrusion detection represented the most common application across all techniques (n=29).

### Distribution of ML Applications Across NIST Cybersecurity Framework Functions

The distribution reveals a pronounced concentration in preincident functions, with Protect, Detect, and Identify collectively accounting for 43 [23-52, 55-67] of the 45 included studies (95.6%). The “Protect” function (22/45, 48.9%) emerged as the most prominent area of ML application, with applications centered on privacy-preserving technologies, data leakage prevention, sensitive information identification, physical isolation mechanisms, asynchronous transfer protocols, and antispoofing measures. Notable among these were privacy-preserving ML techniques, including federated learning approaches. The “Detect” function (13/45, 28.9%) represented the second major application area, encompassing intrusion detection and mitigation, anomaly-based threat detection, spear phishing attack detection, and impact mitigation. The “Identify” function (8/45, 17.8%) showed moderate representation, with studies focusing on risk assessment and prioritization, predictive analytics, threat prediction and analysis, vulnerability detection, and predictive analysis models. These applications demonstrated a proactive approach to cybersecurity through anticipatory capabilities.

**Figure 4.**
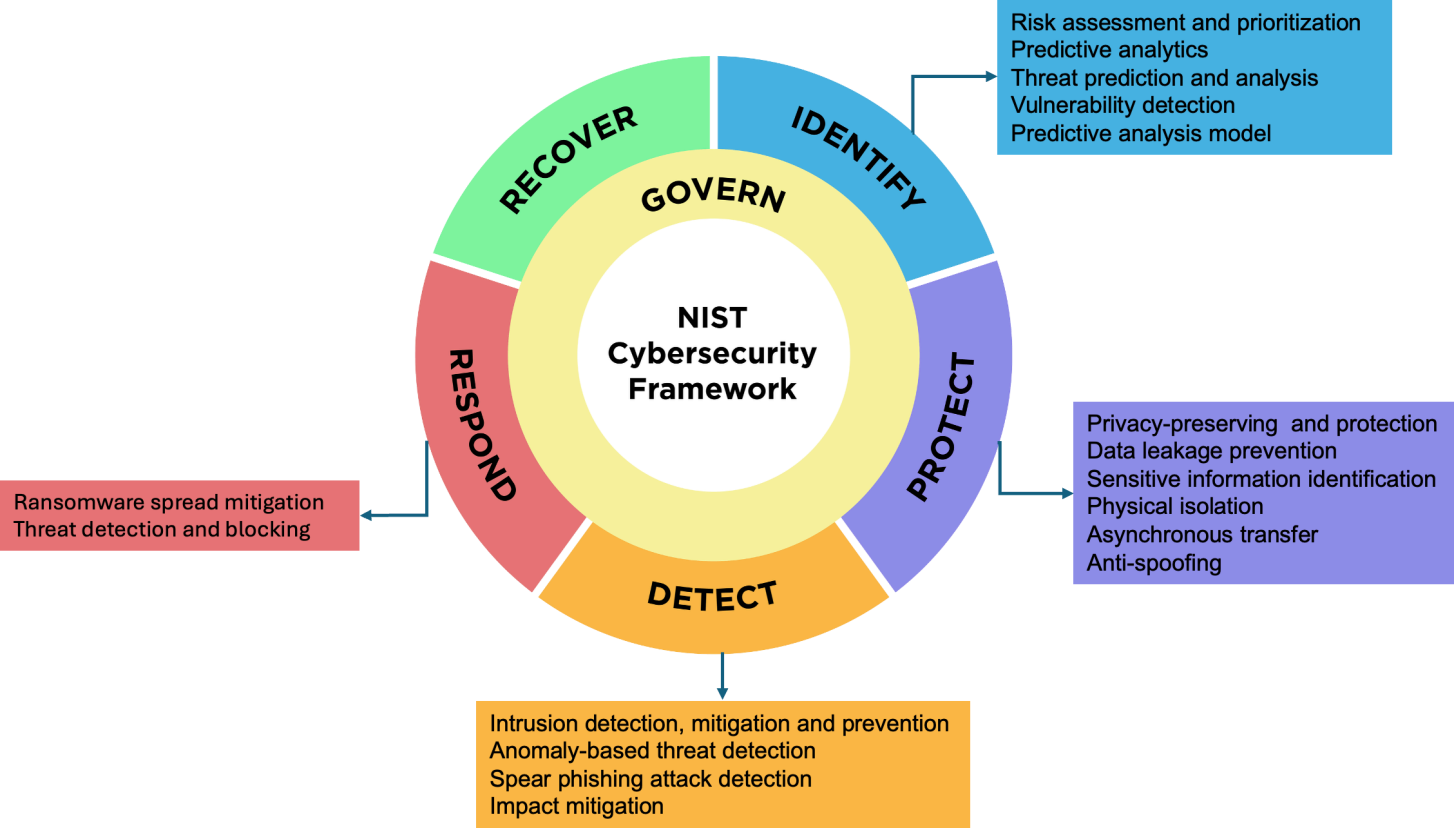
Distribution of 45 peer-reviewed studies examining machine learning–based cyber resilience tools in healthcare settings (2019‐2025) across the six core functions of the National Institute of Standards and Technology Cybersecurity Framework, version 2.0 (NIST CSF 2.0) [16]: Identify, Protect, Detect, Respond, Recover, and Govern. Studies were identified through systematic searches of Ovid MEDLINE, Embase, and Scopus conducted on July 30, 2025. NIST: National Institute of Standards and Technology.

**Figure 5.**
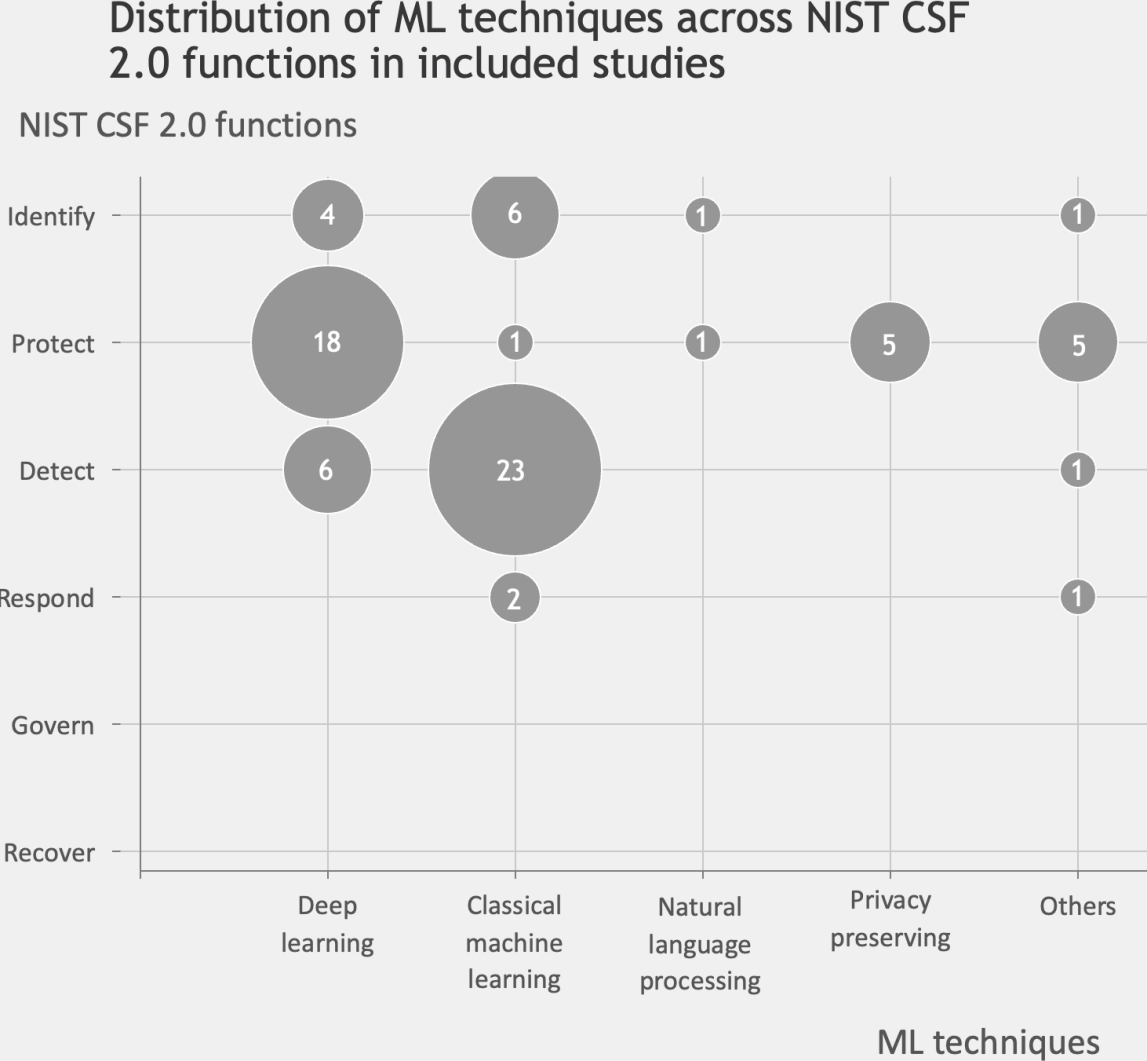
Bubble chart mapping machine learning technique categories against NIST CSF 2.0 (National Institute of Standards and Technology Cybersecurity framework 2.0) functions, with bubble size proportional to the number of included studies (range: 1‐23). Classical machine learning applied to threat detection (n=23) and deep learning applied to protective controls (n=18) dominated the evidence base. No studies addressed the Govern or Recover functions, representing critical gaps in the application of machine learning to health care cybersecurity governance and resilience. CST: Cybersecurity framework; ML: Machine learning; NIST: National Institute of Standards and Technology [16].

The “Respond” function received limited attention, with only two specific applications identified: ransomware spread mitigation and threat detection and blocking. The “Recover” and “Govern” functions received no representation from the reviewed literature (Figure 4). Classical ML and DL dominated across all represented functions, concentrated within Protect and Detect; no ML technique was applied to the Govern or Recover functions (Figure 5). Detailed findings for the studies included in each function are presented in the following sections. Multimedia Appendix 2 [24-68] provides a table that summarizes the included studies with the lead author, year of publication, and main author country of authorship.

### Protect Function

Of the 45 studies, 22 studies [29-39,57-60,62-68] focused on the “Protect” function, collectively encompassing 295,201 patient records, 1,073,101 public data samples, 38,514 discharge summaries, and 100 test instances. The main focus was on privacy-preserving and data protection techniques (n=19), with some studies addressing physical or network isolation and antispoofing measures.

Techniques clustered around cryptographic and decentralized learning approaches include computation on encrypted data [32] using homomorphic encryption (HE) variants, attribute-based schemes, and elliptic-curve methods [29,39,57]. Deidentification pipelines such as Open De-Identification and hybrid NLP or ML approaches that remove or replace sensitive health information with realistic surrogates. Generative models such as generative adversarial networks (GANs), deepGAN [34,37], and local differentially private GANs [68] that produce synthetic medical data unlinked to individuals. Additionally, federated learning (FL), which enables collaborative model training across decentralized data sources without centralizing raw data, is further enhanced by differential-privacy extensions [31,66].

HE enabled computation on encrypted data (including packed [65] and fully HE variants [30] for medical images); FL and decentralized or relay frameworks preserved data sovereignty while supporting collaborative model training [38,59,60]. Ensemble techniques targeted data leakage and integrity (NLP and artificial neural network) [67] and automated phishing detection (Multinomial Naïve Bayes, support vector machine [SVM], convolutional neural network [CNN], long short-term memory [LSTM]) [33]. Network flow watermarking techniques have been used for real-time privacy and data leakage detection methods in digital health care systems, with particular application in medical image protection [58]. ChatGPT-3.5 and GPT-4 (OpenAI) were tested for clinical note deidentification and synthetic data generation [36]. Unlike locally deployed open-weight models, these are application programming interface–based systems without publicly available weights; testing on clinical text therefore involves transmitting potentially identifiable data to third-party infrastructure, a data governance exposure that is absent when using locally hosted alternatives.

Reported outcomes were generally strong, with FL studies commonly reporting accuracies in the 90%‐99% range, under controlled or simulated conditions [31,38,59,62,66]. Open De-Identification and NLP pipelines achieved high precision and *F*_1_-scores (eg, Open De-Identification: precision 95.4%, *F*_1_-score 92.2%) [29,39,57,67,68]. Several GAN or large language model approaches reported accuracies over 82% up to ≈99% [35,37,60,63]. Large-scale phishing detection achieved ≈99.4% accuracy across 525,754 emails, as reported by study authors [33]. Notably, an Open De-Identification pipeline was reported to be deployed in real time at a tertiary hospital and demonstrated more than 90% performance on privacy tasks using real EHR datasets [29]. Reported outcomes suggest strong technical performance for privacy tasks; however, evaluations were heterogeneous, metrics were inconsistently reported, and few studies included external or operational validation.

### Detect Function

There were 13 studies belonging to the “Detect” function. These include a total of 1,070,777 EHR entries, 41,403 attack events, and 777,983 public data records [40-52]. Cyber resilience techniques include intrusion detection (n=8), intrusion detection with mitigation or prevention (n=2), and other single-instance methods such as anomaly, insider-threat, spear phishing, and impact-mitigation detection.

Classical ML techniques such as SVM, decision tree (DT), random forest (RF), K-nearest neighbor (KNN), Naïve Bayes, logistic regression, and gradient-boosted learners such as extreme gradient boosting (XGBoost) or adaptive boosting [42,44,49,50] formed the backbone of many studies and were used to detect EHR data misuse and to classify different attack types. Ensemble and hybrid schemes combined multiple classical learners (eg, a combination of SVM, KNN, DT, or RF ensembles) and reported that ensemble approaches delivered competitive accuracy with added stability across attack types in simulated evaluations [45]. DL Architectures (CNNs, LSTMs, gated recurrent units) and bespoke neural networks such as ImmuneNet and hybrid deep frameworks predominated where high-dimensional or sequential inputs were required (network flows, email text, and time-series vitals) [40,47,48]. These models frequently produced near-perfect benchmark scores (many reports more than 99% accuracy on public datasets under controlled testing conditions) at the cost of greater computational complexity. Unsupervised and semisupervised methods (isolation forest, local outlier factor, and other anomaly detectors) were used either standalone for novelty detection or as prelabeling steps to bootstrap supervised training; studies that combined unsupervised labeling with downstream classifiers showed strong sensitivity for contextual anomalies [41,45]. Studies also explored specialized multimodal fusion architectures integrating CNN, LSTM, or gated recurrent units feature streams for richer representation detection rate on the publicly available dataset, and bio-inspired or swarm-intelligence methods (eg, swarm-based network watermarking) for robust, low-overhead detection and real-time data-leakage protection [52].

### Identify Function

There were eight studies in the “Identify” function, including 352 cyberattack events, 51 expert annotations, 514,220 NLP tokens, 1000 execution paths, 65,536 network packets, and 825 county-level data points [24-28,55,56,61]. Studies focused primarily on risk assessment (n=5) and predictive analysis (n=3).

Studies demonstrated ML applications across cybersecurity risk assessment and vulnerability prediction in health care settings. Predictive models for hospital data breaches and security vulnerability assessment and their potential to evolve into usable exploits were developed from classical learners (KNN, SVM, DT, RF) [25,55,56]. Probabilistic approaches such as Bayesian networks [28] and fuzzy or knowledge-based systems (adaptive neuro-fuzzy inference system) [61] were applied to security risk assessment during health care web application development, reporting strong performance on the datasets evaluated. Deep neural networks (stacked autoencoders and deep neural networks) developed a hospital computer network information security risk assessment [26,28]. An intelligent vulnerability detector for healthcare cyber-physical systems used recurrent neural network/LSTM architectures to identify vulnerabilities [27]. NLP pipelines were applied to large text corpora to extract threat and vulnerability intelligence [26]. However, these studies were simulation-based, used heterogeneous datasets, and inconsistently reported evaluation metrics, limiting cross-study comparability. Where provided, point estimates included KNN ≈87% (prediction accuracy), a top NLP study reporting 99.8% accuracy (with precision 96.6% and *F*_1_-score 87.5%), a Bayesian network reporting 75% accuracy (recall 73%), and an SVM model achieving 83.1% accuracy (precision 65.1%, recall 58.3%, and *F*_1_-score 61.5%).

### Respond Function

A total of Two studies belonged to the “Respond” function of the NIST-CSF 2.0. One study explored ransomware spread mitigation involving 150,537 ransomware datasets, and the other focused on intrusion mitigation using 16,000 patient vital signs [53,54]. A real-time ransomware detection system used SVM for anomaly detection and Naïve Bayes for classification. Integrating software-defined networking and network function virtualization, it isolated and replaced infected systems, mitigating attacks in under 30 seconds, faster than the typical 1-minute spread [53]. The second study proposed a crossover-based multilayer perceptron model–detected attacks on EHR and promptly alerted health care professionals to prevent data leakage. Experimental results on synthetic and real-world datasets showed the crossover-based multilayer perceptron model outperformed existing methods using DL and CNN, with 97% accuracy, 93% precision, 92% recall, and 92% *F*_1_-score, while also exhibiting lower computational complexity [54].

## Discussion

### Principal Findings

This review maps ML-driven cybersecurity applications in health care against the NIST CSF 2.0 to identify where evidence clusters and gaps persist. In doing so, it provides the first structured, health care–specific synthesis of ML cybersecurity evidence aligned to this framework. Classical algorithms, DL, NLP, and FL were the predominant technique categories, applied across diverse health care environments internationally, with research output accelerating sharply from 2022 [69-71]. The evidence base is heavily concentrated in preincident functions, with Protect, Detect, and Identify collectively accounting for the substantial majority of included studies, while Respond was underrepresented; Recover and Govern remain entirely absent. Despite high technical accuracy under controlled conditions, real-world deployment was almost entirely absent, with barriers around dataset generalizability, explainability, and regulatory readiness persisting across the literature. These findings have direct implications for how health care organizations might prioritize ML adoption.

### Overall Contribution and Landscape of Included Studies

Prior reviews by Wiafe et al [17], Ali et al [18], and Dhanushkodi and Thejas [20] examined ML and AI for cybersecurity across general computing environments, cataloging techniques such as intrusion detection, malware analysis, and anomaly detection, but none were specific to health care or mapped findings against an established governance framework [17-20]. The global landscape of medical AI has evolved rapidly, with China emerging as a leading contributor in health care AI research, surpassing the United States in the number of publications over the past two years [72]. Nonetheless, this review also likely underestimates China’s contributions because of the language limitations in the search strategy.

This growth in research output reflects a scientific response to an expanding threat landscape, further accelerated by the COVID-19 pandemic, which drove rapid digitization of mission-critical health care services [73,74]. The shift to interconnected health infrastructure, telehealth, and widespread adoption of connected medical devices expanded the attack surface. The escalation of cyberattacks from 2022 onward, fueled by the high value of patient data for identity theft and insurance fraud, further intensified the urgency for ML-driven solutions [75,76]. Health care organizations have been identified as prime targets for financially motivated attackers, partly because the criticality of patient care services creates pressure to restore operations rapidly [77].

The dominance of the “Protect” function is consistent with the heightened regulatory and ethical scrutiny surrounding patient data in health care, where data protection obligations create strong institutional incentives for this class of solution [78]. The prominence of the Detect function likely reflects the technical tractability of intrusion detection as a well-defined, benchmarkable problem with established datasets [79]. By contrast, the near-absence of Respond and the complete absence of Recover and Govern studies suggest that the research literature has not yet engaged substantively with the organizational dimensions of cyber resilience through ML approaches [2,16,69].

Across the broader cybersecurity literature, governance tasks such as risk monitoring, automated compliance, and auditability have received comparatively limited attention relative to detection and protection [80,81]. On the other hand, studies in other critical infrastructures have demonstrated automated root cause analysis and self-healing mechanisms achieving zero-downtime recovery [82], highlighting the translational opportunity for health care. Governance and risk management are therefore essential to guide the responsible deployment of ML models and organizational resilience [16].

### Performance of ML Techniques

DL and hybrid models consistently demonstrated strong detection performance in controlled evaluations, with CNN and LSTM architectures predominating, consistent with the systematic review by Ali et al [18], though results derive predominantly from synthetic or benchmark datasets and lack operational validation. Similarly, classical learners (RF, DT, and SVM) remained competitive in controlled evaluations, though real-world deployment performance was not assessed in the included studies [40,41,47-49]. Supervised ML algorithms, particularly ensemble SVMs and ANNs, have dominated academic literature. Similar findings were reported by Mukkamalla et al [83]. Likewise, the broader mapping study by Wiafe et al [17] identified rising SVM usage between 2013 and 2018, attributed to their suitability for small, high-dimensional, and nonlinear datasets.

Ensemble deidentification approaches, HE combined with other ML models, and FL were the most frequently adopted techniques. FL allows different institutions to train ML models on their local data and share only model parameters with a central server or other nodes, rather than sensitive raw data [31,66,84]. FL has been proposed as particularly suited to dynamic health care networks because it enables real-time updates and scales across institutions without raw data sharing. However, challenges such as institutional data heterogeneity, communication costs, model aggregation vulnerabilities, and potential biases limit real-world reliability [85]. These aggregation vulnerabilities include gradient reconstruction attacks, in which shared model updates are exploited to recover original training data, and model poisoning, in which malicious local updates corrupt the global model [86,87]. Combining FL with HE strengthens the privacy-preserving architecture, as model updates remain encrypted during transfer [88].

Proactive risk assessment applications demonstrated that both data-driven and knowledge-based ML approaches can identify vulnerabilities and predict breaches in health care settings, though all evaluations were simulation-based and none approached operational readiness [25,26,61]. In health care network environments, where the vast majority of traffic is benign, class imbalance means that high accuracy can be achieved even by models that fail to detect most attacks [89]. Metrics such as precision, recall, and *F*_1_-score provide a more complete picture, but these too were inconsistently reported across the included studies [81]. The asymmetric cost of errors is particularly consequential in clinical settings: false negatives carry direct patient safety implications, while high false positive rates risk alert fatigue among already stretched clinical and information technology teams, potentially causing genuine threats to be overlooked [90]. The absence of standardized, clinically contextualized evaluation frameworks across the included studies therefore limits the extent to which reported performance figures can be used to judge real-world effectiveness [91].

### Barriers to Real-World Applicability

Many studies relied on outdated or non–health care network datasets (CICIDS-2017, NSLKDDCup99, Kaggle), with the oldest exceeding over 20 years old [92]. Methodological inconsistencies, including nonstandardized metrics, varying preprocessing techniques, undocumented hyperparameter tuning, and an absence of benchmarking against established cybersecurity tools, further undermine reproducibility [81,89].

These patterns have substantive implications for the maturity and reliability of the evidence base. First, the near-exclusive reliance on synthetic or benchmark datasets raises questions about generalizability, as models optimized on static, historical network traffic data are unlikely to perform reliably against the dynamic, heterogeneous threat environments of modern health care infrastructure [92]. Second, the absence of external or prospective validation means that reported performance figures cannot currently be taken as indicators of deployment readiness; high benchmark accuracy is a necessary but insufficient condition for clinical adoption without validation on representative, real-world data [89,91]. Third, heterogeneous evaluation practices, including inconsistent metric selection, varying dataset splits, and undocumented tuning, make cross-study comparison unreliable and limit the conclusions that health care organizations or policymakers can draw [81,89]. The evidence base is therefore best characterized as exploratory rather than confirmatory. It demonstrates that ML techniques can achieve strong results under controlled conditions but are not yet sufficient to establish deployment readiness in clinical environments.

On an institutional level, health care organizations lack the technical infrastructure and interdisciplinary expertise to implement and maintain ML-driven cybersecurity systems effectively [90,93]. This challenge is compounded by the fact that explainable AI, intended to make AI understandable, remains poorly defined, with terms like interpretability and transparency often used interchangeably [94]. Explainable AI has been proposed to build clinician trust, support regulatory approval, and improve communications between clinicians and cybersecurity teams [95]. Several studies noted the absence of explainability as a barrier to clinical trust, pointing to explainable AI as a potential avenue for future research, though its practical integration into health care cybersecurity remains undemonstrated in the included literature [30,48].

Ethical, regulatory, and infrastructural challenges hinder ML adoption in health care [96]. Access to sensitive patient data raises concerns around privacy, consent, and compliance with regulations [15,97,98]. Rapid development and simulation of ML models have outpaced regulatory frameworks, creating a gray area that increases compliance burdens and delays clinical deployment. Recent policy initiatives, including the National Health Service Innovation Accelerator [99] and the US AI Action Plan [15], signal growing governmental recognition of AI’s role in health care cybersecurity and could provide implementation pathways for organizations navigating this landscape. Parallel efforts from the European Commission’s hospital cybersecurity action plan and the WHO’s AI for health framework similarly reflect an emerging international consensus on AI-enabled cyber resilience in health care [100,101].

### Limitations of This Review

This review has several limitations. First, language and publication bias may have excluded non-English or nonindexed studies, and gray literature was not systematically searched. Second, the rapid evolution of ML and cyber threats means findings become outdated within a short timeframe. Third, heterogeneity in study designs, datasets, and evaluation metrics limited direct cross-study comparison. Fourth, the review excluded medical devices to focus on institutional cybersecurity, potentially omitting insights into device-specific risks, and proprietary AI-based cybersecurity products were not included due to limited methodological transparency, which may restrict understanding of current real-world implementations. Additionally, classification of studies against NIST CSF 2.0 functions was conducted by the review team and, while guided by each study’s primary cybersecurity objective, remains inherently interpretive. Studies addressing multiple cybersecurity tasks may reasonably be mapped to more than one function, and alternative classifications by other reviewers are possible. Finally, the search was conducted till July 30, 2025, as prespecified in the registered protocol (OSF Registries 4cjmu); studies published after this date are not captured, and an updated review is recommended within two years given the pace of publication in this field.

### Future Directions and Operational Implications

Health care organizations face the dual challenge of implementing ML-driven cybersecurity while managing legacy infrastructure, limited budgets, and regulatory uncertainty. Current national and international cybersecurity strategies consistently call for AI-driven detection, privacy-preserving architectures, and governance frameworks, yet this review demonstrates that the research base supporting the latter two remains markedly underdeveloped [14,15,99].

Based on the distribution of evidence identified in this review, a set of priorities for pilot testing can be outlined for health care organizations. These priorities are presented as hypotheses for consideration rather than a validated deployment model. The relative maturity of evidence in the “Detect” function, with intrusion detection representing the most studied application, represents a more tractable starting point for organizations considering ML pilot testing [40-42,44,45,47-50,52]. However, as Heine et al [91] highlight, despite excellent results on benchmark datasets, ML-based intrusion detection systems remain difficult to deploy in practice, with current simulation-based evaluation methodologies poorly reflecting real-world performance.

The growing volume of studies addressing privacy-preserving techniques, particularly FL and HE, indicates an emerging evidence base that may support a subsequent pilot-testing priority, particularly for organizations managing multisite data under strict privacy regulation, though operational implementation evidence remains sparse [31,66,92]. The absence of any studies addressing the Govern or Recover functions represents the most significant gap identified by this review, underscoring that governance frameworks and recovery protocols for ML-driven cybersecurity in health care remain open research priorities rather than established practice.

The absence of real-world implementation evidence has direct resource implications for health care organizations considering ML adoption. These span AI procurement, infrastructure upgrades, staff training, and ongoing model monitoring, maintenance, and external validation. Pilot programs with careful cost tracking, rigorous documentation, and a primary focus on safety are needed to bridge the gap between experimental research and operational deployment. Without this foundation, policy ambition will continue to outpace the evidence needed to deploy ML-driven cybersecurity safely in clinical environments. The gap between policy ambition and operational evidence is unlikely to remain static. Anticipated advances in quantum computing represent a longer-term threat vector with the potential to render current cryptographic protections obsolete, reinforcing the case for sustained investment in health care–specific cybersecurity research and the development of adaptive defensive architectures [102-104].

### Conclusions

By mapping ML-driven cybersecurity in health care against all 6 functions of the NIST CSF 2.0, this review offers a governance-aligned synthesis that complements prior reviews organized by attack type or ML architecture [17-20,105]. The NIST CSF 2.0 framework, commonly used to evaluate organizational cyber resilience, makes findings translatable into implementation priorities for organizations. Evidence clusters in Identify, Protect, and Detect; Respond, Recover, and Govern remain unaddressed; and real-world deployment was almost entirely absent. These later functions precisely determine whether a health care organization can contain, recover from, and build lasting organizational cyber resilience from an attack.

The relative maturity of intrusion detection and privacy-preserving methods offers a defensible entry point for ML adoption, but organizations pursuing end-to-end cyber resilience cannot yet draw on a validated evidence base for governance or recovery. Until sustained investment in governance research and real-world validation with representative datasets addresses these gaps, ML in health care cybersecurity will remain a promising experimental tool rather than a trusted operational safeguard.

## Supplementary material

10.2196/93950 Multimedia Appendix 1 Detailed search strategy, inclusion and exclusion criteria.

10.2196/93950 Multimedia Appendix 2 Supplementary tables outlining data charting.

10.2196/93950 Checklist 1 PRISMA-ScR Checklist.

10.2196/93950 Checklist 2 PRISMA-S checklist.

## Abbreviations

CNN: convolutional neural network
DL: deep learning
DT: decision tree
EHR: electronic health record
FL: federated learning
GAN: generative adversarial network
HE: homomorphic encryption
KNN: K-nearest neighbor
LSTM: long short-term memory
ML: machine learning
NIST CSF 2.0: National Institute of Standards and Technology Cybersecurity Framework 2.0
NLP: natural language processing
PRISMA-S: Preferred Reporting Items for Systematic Reviews and Meta-Analyses literature search extension
PRISMA-ScR: Preferred Reporting Items for Systematic Reviews and Meta-Analyses extension for Scoping Reviews
RF: random forest
SVM: support vector machine
XGBoost: extreme gradient boosting
