# Supplementary material for: Mapping Machine Learning–Driven Cybersecurity Solutions in Health Care: Scoping Literature Review
Source: J Med Internet Res. 2026 Jul 27;28:e93950. doi: 10.2196/93950 (PMC13405368; doi:10.2196/93950)
Supplement: Multimedia Appendix 1 [file jmir-v28-e93950-s001.docx]

# Search and Screening Strategy

Title: Mapping Machine Learning Driven Cybersecurity Solutions in Healthcare: A Scoping Literature Review

Aim: This scoping review aims to address this gap by synthesising current evidence on the use of ML to enhance cybersecurity in healthcare, identifying existing approaches, evaluating their effectiveness, and outlining key directions for future research and implementation.

## PICO Format

**Population**: Studies involving healthcare systems, such as hospitals, clinics, and health networks, with a focus on organisational-level cybersecurity.

**Intervention**: ML-based approaches that contribute to cyber resilience

**Comparison**: NA

**Outcome**: Assessment of use of AI for data privacy, protection of healthcare data, and the strengthening of cybersecurity practices.

**Study Design**: Peer reviewed studies with experimental studies utilising ML for cyber-resilience in healthcare.

## Search Strategy

| Ovid MEDLINE(R) ALL <1946 to July 30, 2025>  1 exp Artificial Intelligence/ 279247  2 exp Neural Networks, Computer/ 100490  3 exp Machine Learning/ 120669  4 Random Forest/ 905  5 Support Vector Machine/ 13288  6 (neural network* or "convolutional neural network*" or CNN or "recurrent neural network*" or RNN or LSTM or GRU or "deep neural network*" or DNN or autoencoder*).ti,ab. 157597  7 ("support vector machine*" or SVM or "support vector classifier*" or SVC or "random forest*" or "decision tree*" or "k-nearest neighbour*" or "k-nearest neighbor*" or KNN or "naive Bayes" or "Bayesian network*" or "gradient boost*" or XGBoost or AdaBoost).ti,ab. 116891  8 ("generative adversarial network*" or GAN or "federated learning" or "natural language processing" or "large language model*" or LLM or BERT or transformer* or "anomaly detection" or "intrusion detection").ti,ab. 60500  9 ("supervised learning" or "unsupervised learning" or "reinforcement learning" or "semi-supervised learning" or "ensemble learning" or "explainable artificial intelligence" or XAI or "explainable AI").ti,ab. 27180  10 Natural Language Processing/ 9253  11 (artificial intelligence or "machine intelligence" or "machine learning" or "deep learning" or "transfer learning").ti,ab. 310746  12 1 or 2 or 3 or 4 or 5 or 6 or 7 or 8 or 9 or 10 or 11 590920  13 exp Computer Security/ 11229  14 exp Privacy/ 18918  15 (cybersecurit* or "cyber security" or "cyber resilience" or cyberresilien* or "cyber defence" or "cyber defense").ti,ab. 2085  16 (ransomware or malware or "denial of service" or DDoS or phishing or "spear phishing").ti,ab. 1460  17 ("intrusion detection" or "anomaly detection" or "threat detection" or "attack detection" or "insider threat*" or "zero-day" or "zero day").ti,ab. 4686  18 ("data breach*" or "data leak*" or "data exfiltration" or "privacy preserving" or "privacy-preserving" or "de-identification" or "deidentification" or "homomorphic encryption" or "differential privacy").ti,ab. 4120  19 ("access control" or "federated learning" or "network security" or "cyber attack*" or "cyberattack*").ti,ab. 5264  20 (((cyber or network* or computer*) adj2 (securit* or attack* or threat* or vulnerab* or breach* or resilien* or incident*)) or "vulnerability assessment" or "penetration test*").ti,ab. 5499  21 exp Delivery of Health Care/ 1455463  22 exp Hospitals/ 341068  23 exp Electronic Health Records/ 35298  24 (healthcare or "health care" or hospital* or clinic* or "health system*" or "health network*" or "health service*").ti,ab. 8073796  25 ("electronic health record*" or EHR or "electronic medical record*" or EMR or "patient record*" or "health informatics" or "clinical information system*").ti,ab. 105745  26 ("picture archiving" or PACS or SCADA or "medical imaging system*" or "health data" or "patient data" or "medical data").ti,ab. 59990  27 (NHS or "National Health Service" or "health infrastructure" or "health organisation*" or "health organization*" or "health institution*").ti,ab. 178083  28 13 or 14 or 15 or 16 or 17 or 18 or 19 or 20 43598  29 21 or 22 or 23 or 24 or 25 or 26 or 27 9050110  30 12 and 28 and 29 3277  31 limit 30 to (english language and yr="2019-2025") 2521 |
| --- |

| Embase Classic+Embase <1947 to 2025 Week 31>  1 exp artificial intelligence/ 198569  2 exp machine learning/ 736734  3 neural network/ 94  4 deep learning/ 113034  5 federated learning/ 1556  6 natural language processing/ 20789  7 (artificial intelligence or "machine learning" or "deep learning" or "transfer learning").ti,ab. 369274  8 (neural network* or "convolutional neural network*" or CNN or "recurrent neural network*" or RNN or LSTM or GRU or "deep neural network*" or DNN or autoencoder*).ti,ab. 184857  9 ("support vector machine*" or SVM or "support vector classifier*" or SVC or "random forest*" or "decision tree*" or "k-nearest neighbour*" or "k-nearest neighbor*" or KNN or "naive Bayes" or "Bayesian network*" or "gradient boost*" or XGBoost or AdaBoost).ti,ab. 149036  10 ("generative adversarial network*" or GAN or "federated learning" or "natural language processing" or "large language model*" or LLM or BERT or "anomaly detection" or "intrusion detection").ti,ab. 49032  11 ("supervised learning" or "unsupervised learning" or "reinforcement learning" or "semi-supervised learning" or "ensemble learning" or "explainable artificial intelligence" or XAI or "explainable AI").ti,ab. 30831  12 1 or 2 or 3 or 4 or 5 or 6 or 7 or 8 or 9 or 10 or 11 971552  13 exp computer security/ 13647  14 exp data privacy/ 4050  15 exp information security/ 3175  16 (cybersecurit* or "cyber security" or "cyber resilience" or cyberresilien* or "cyber defence" or "cyber defense").ti,ab,kw. 2377  17 (ransomware or malware or "denial of service" or "DoS attack*" or DDoS or phishing or "spear phishing" or "social engineering" or "man-in-the-middle").ti,ab,kw. 1861  18 ("intrusion detection" or "anomaly detection" or "threat detection" or "attack detection" or "insider threat*" or "zero-day" or "zero day").ti,ab,kw. 5428  19 ("data breach*" or "data leak*" or "data exfiltration" or "privacy-preserving" or "de-identification" or "deidentification" or "re-identification" or "homomorphic encryption" or "differential privacy").ti,ab. 5712  20 ("homomorphic encryption" or "federated learning" or "network security" or "cyber attack*" or "cyberattack*").ti,ab. 4342  21 (((network* or computer* or cyber or electronic*) adj2 (securit* or vulnerab* or threat* or breach* or attack*)) or cybersecurit* or cyberresilien* or "vulnerability assessment").ti,ab,kw. 6221  22 exp health care/ 8217876  23 exp hospital/ 1828106  24 exp electronic health record/ 75720  25 exp telemedicine/ 97451  26 (healthcare or "health care" or hospital* or clinic* or "health system*" or "health network*" or "health service*").ti,ab,kw. 12370510  27 ("electronic health record*" or EHR or "electronic medical record*" or EMR or "patient record*" or "health informatics" or "clinical information system*").ti,ab,kw. 213348  28 ("picture archiving" or "medical imaging system*" or "health data" or "patient data" or "medical data" or telemedicine or telehealth).ti,ab,kw. 152769  29 (NHS or "National Health Service" or "health infrastructure" or "health organisation*" or "health organization*" or "health institution*").ti,ab,kw. 254216  30 13 or 14 or 15 or 16 or 17 or 18 or 19 or 20 or 21 34178  31 22 or 23 or 24 or 25 or 26 or 27 or 28 or 29 16782521  32 12 and 30 and 31 6078  33 limit 32 to (english language and human and yr="2019 - 2025") 4526 |
| --- |

| Scopus (30^th^ July 2025) 3300 articles |
| --- |
| TITLE-ABS ( ( "artificial intelligence" OR "machine learning" OR "deep learning" OR "supervised learning" OR "unsupervised learning" OR "reinforcement learning" OR "semi-supervised learning" OR "ensemble learning" OR "explainable artificial intelligence" OR "explainable AI" OR "neural network*" OR "convolutional neural network*" OR "recurrent neural network*" OR "deep neural network*" OR LSTM OR GRU OR DNN OR RNN OR CNN OR "generative adversarial network*" OR GAN OR "federated learning" OR "natural language processing" OR "large language model*" OR LLM OR BERT OR transformer* OR "anomaly detection" OR "intrusion detection" OR "support vector machine*" OR SVM OR "support vector classifier*" OR SVC OR "random forest*" OR "decision tree*" OR "k-nearest neighbour*" OR "k-nearest neighbor*" OR KNN OR "naive Bayes" OR "Bayesian network*" OR "gradient boost*" OR XGBoost OR AdaBoost ) AND ( cybersecurit* OR "cyber security" OR "cyber resilience" OR "cyber defence" OR "cyber defense" OR "cyber attack*" OR "cyberattack*" OR ransomware OR malware OR phishing OR "spear phishing" OR "denial of service" OR DDoS OR "intrusion detection" OR "anomaly detection" OR "threat detection" OR "attack detection" OR "insider threat*" OR "zero-day" OR "data breach*" OR "data leak*" OR "data exfiltration" OR "privacy preserving" OR "privacy-preserving" OR "de-identification" OR "deidentification" OR "homomorphic encryption" OR "differential privacy" OR "access control" OR "network security" OR "vulnerability assessment" OR ( cyber W/2 ( securit* OR attack* OR threat* OR vulnerab* OR breach* OR resilien* OR incident* ) ) OR ( network* W/2 ( securit* OR attack* OR threat* OR vulnerab* OR breach* OR incident* ) ) OR ( computer* W/2 ( securit* OR attack* OR threat* OR vulnerab* OR breach* OR incident* ) ) ) AND ( healthcare OR "health care" OR hospital* OR clinic* OR "health system*" OR "health network*" OR "health service*" OR "electronic health record*" OR EHR OR "health data" OR "patient data" OR "medical data" OR NHS OR "National Health Service" ) ) AND PUBYEAR > 2018 AND PUBYEAR < 2026 AND ( LIMIT-TO ( PUBSTAGE , "final" ) ) AND ( LIMIT-TO ( DOCTYPE , "ar" ) ) AND ( LIMIT-TO ( LANGUAGE , "English" ) ) |

## Inclusion Criteria

- Studies involving **healthcare systems**, such as hospitals, clinics, and health networks, with a focus on **organisational-level cybersecurity**.
- ML-based approaches that contribute to **cyber resilience**, including but not limited to **predictive analytics, anomaly detection**, and **automated response systems**.
- Articles that assessed the use of AI for **data privacy, protection of healthcare data**, and the strengthening of cybersecurity practices.
- **Experimental and observational studies** that involved the **development, validation, or comparative evaluation** of AI models.
- **Publication Type:** Articles published in **peer-reviewed journals** with **full-text availability in English**.

## **Exclusion Criteria**

- Articles **lacking a clearly documented AI/ML model** or algorithmic approach.
- Studies primarily focused on **Internet of Medical Things (IoMT)**, **smart/wireless healthcare devices**, or **blockchain-based cybersecurity**.
- Literature that **did not examine AI/ML's impact on cybersecurity** at an organisational level in healthcare.
- **Non-peer-reviewed** sources and non-English publications, including **conference abstracts, books, editorials, commentaries, case reports**, and **review articles**.
- **Theoretical or technical concept papers** that did not include empirical data or evaluation.
