# Supplementary material for: Mapping Machine Learning–Driven Cybersecurity Solutions in Health Care: Scoping Literature Review
Source: J Med Internet Res. 2026 Jul 27;28:e93950. doi: 10.2196/93950 (PMC13405368; doi:10.2196/93950)
Supplement: Multimedia Appendix 2 [file jmir-v28-e93950-s002.docx]

| **Table 1. Studies Addressing the ‘Identify’ Function of the NIST Cybersecurity Framework (NIST-CSF 2.0)** | | | | | | | |
| --- | --- | --- | --- | --- | --- | --- | --- |
| **Lead Author (Year)** | **Country of Origin** | **Aim of Cyber Resilience** | **Healthcare settings** | **AI Model Methodology** | **Training Data & Source** | **Sample Size** | **Key Findings and Outcomes** |
| Hüseyin Ünözkan, (2022) [1] | Turkey | Risk Assessment, Predictive Analytics | Healthcare systems | ANN, KNN | Real-life cyberattacks dataset | 352 cyberattacks | Identified as the most successful model for predicting cyberattacks on healthcare IT systems, achieving a high success rate of 87.3%. |
| Jasleen Kaur (2020) [2] | India | Risk Assessment, Threat Prediction | Healthcare Systems | ANFIS & Fuzzy logic | Expert knowledge via Questionnaires | 51 experts | This superior performance was validated by the ANFIS technique achieving exceptional reliability metrics, including a correlation coefficient (R) of 1.0000 for the assessment of all eight individual security risk factors |
| Stefano Silvestri, (2023)[3] | Italy | Risk Assessment | Cyber-Physical Systems | NLP | Public cybersecurity datasets (Hacker News, CVE reports, MITRE, OSINT) | 514,220 tokens | The experiments confirmed that the SecBERT model provided a slight performance boost for Named Entity Recognition (NER) (0.87 F1 score) and that XGB performed better than logistic regression in predicting vulnerability levels based on CVE reports (MAE of 0.9326, MSE of 2.4744, and R2 of 0.0956) |
| Guizhen Li, (2024)[4] | China | Risk Assessment | Healthcare Networks | DNN | Real-world expert assigned scores | - | The experimental results showed that the method's assessment indices mainly converged between 20 and 40 (Level 2 security), which aligns appropriately with the known high security status of the research hospital's updated network system. |
| Shareeful Islam, (2022)[5] | UK | Threat Analysis, Risk Prioritization | Supply Chain | DT & RF | Public cybersecurity datasets (CVE, CVSS3.1, NVD) | - | ML algorithms increase the accuracy of vulnerability exploitability prediction. Among the models tested for predicting vulnerability exploitability using the CVE dataset, the Random Forest (RF) algorithm provided the highest accuracy at 63%, slightly outperforming Decision Tree (62%) and Linear Regression (61%) |
| Xiaoyu Yi, (2023)[6] | China | Vulnerability Detection | Cyber Physical Systems | RNN, LSTM | Real-world software | 1000 execution paths | Achieved a vulnerability recognition accuracy of more than 95%, demonstrating superior performance compared to other baseline models like Random Forest, MLP, and SVM |
| Mohammad H M Yusof, (2022)[7] | Malaysia | Predictive Analysis Model | Healthcare infrastructure | BN | KDD Cup 99 dataset | 65,536 packets | Outperformed other classification models (including SVM and KNN) in detecting attacks on healthcare infrastructure, achieving a 100% detection rate and 86% accuracy with a low False Alarm Rate (FAR) of 14% on ground truth testing datasets. The predictive model also demonstrated the critical capability of accurately detecting a zero-day attack on the healthcare provider's network months prior to its actual occurrence. |
| Diane Dolezel, (2023)[8] | USA | Risk assessment | Healthcare Systems | SVM | Real-world hospital and county level data (Definitive Healthcare, US Census Bureau, Bureau of Labor Statistics) | 1032 counties | The SVM model performing the classification task best compared to logistic regression and perceptron models. Specifically, the highest odds ratios, indicating a higher risk of breach, were associated with factors such as inpatient workload, medical centre status, paediatric trauma centre status, and accounts receivable. |

| **Table 2. Studies Addressing the ‘Protect’ Function of the NIST Cybersecurity Framework (NIST-CSF 2.0)** | | | | | | | |  |
| --- | --- | --- | --- | --- | --- | --- | --- | --- |
| **Lead Author** | **Country of Origin** | **Aim of Cyber Resilience** | **Healthcare settings** | **AI Model Methodology** | **Training Data & Source** | **Sample Size** | **Key Findings and Outcomes** | |
| Hansle Gwon, (2024)[9] | Republic of Korea | Privacy-preserving, prevent data leakage | Electronic Health Records | LDP-GAN (NLP & ANN) | Real-world hospital EHR dataset | 6625 patients | Demonstrated greater stability and linear change in utility and privacy responses concerning the privacy budget (ϵ) compared to DP-GAN. LDP-GAN was generally more secure against attacks and exhibited a more stable and efficient trade-off relationship, particularly showing higher utility than DP-GAN when the noise was strong (low ϵ) | |
| Guilherme Goldschmidt, (2023)[10] | Brazil | Data Leakage Prevention, Sensitive Information Identification | Electronic Health Records | NLP & ANN | Real-world hospital EHR dataset (Porto Alegre, Brazil) | - | Yielding a macro-average Precision of 98.0%, Recall of 86.0%, and F1-Score of 91.0% on the test set, alongside an overall accuracy of 95%. This machine learning approach was more effective than the best evaluated SpaCy approach (based on ANN), which recorded a macro-average F1-Score of 80.92%. | |
| Tongyuan Huang, (2023)[11] | China | Privacy-preserving | Medical images | DNN | Real medical image Kaggle database | - | The scheme successfully maintained all Normalized Correlation Coefficient values above 0.8 under various geometric and common attacks, showing a maximum performance improvement of approximately 30% against geometric attacks compared to existing methods | |
| Mahmuda Akter, (2022)[12] | Australia | Privacy-Preserving | Healthcare Systems | FL, CNN | MNIST | 70,000 samples | Employed Edge Intelligence and differential privacy for smart healthcare systems, consistently outperformed the baseline method (no edge aggregator) in terms of higher testing accuracy and lower training error across various datasets and distribution methods. The proposed framework achieved a high testing accuracy of 90% and a high privacy rate, demonstrating better overall performance than the baseline technique | |
| Zhiwei An, (2023)[13] | China | Privacy-Preserving | Healthcare Systems | FL, CNN | MNIST and UCI Smartphone dataset | 10,299 samples | Significantly outperformed baseline federated learning models across all tested non-i.i.d. datasets. This superior performance was validated by achieving the highest accuracy metrics, such as 0.970 on MNIST and 0.941 on UCI datasets. | |
| Nasibeh Mohammadi, (2024)[14] | Iran | Privacy-Preserving | Healthcare Systems | FL, DRNN, LSTM, GRU | Kaggle, Amazon UCI, Conditional datasets | 798,832 samples | Results confirmed that the accuracy (99%) of the access control system was consistently higher across all tested datasets after knowledge aggregation via federated learning compared to the accuracy achieved by the individual local systems before aggregation | |
| Jafar A. Alzubi, (2022)[15] | Jordan | Privacy-Preserving | Medical Data | DNN | Real-world sleep stage dataset (University of Dublin) | 75,128 samples | The PPEDL-MDTC technique resulted in a maximal detection accuracy of 0.9813 on the activity recognition dataset and 0.9650 on the UCD Sleep Stage dataset, outperforming recent comparative models | |
| José Cabrero-Holgueras, (2023)[16] | Spain | Privacy-Preserving | Medical Data | CNN | Real-world CheXpert dataset | 250 patients | Demonstrated that transformations required to interconnect layers (Initial Representation and Result Transformation) pose a significant overhead, which must be accounted for in DL architecture design to optimize inference performance over encrypted data. | |
| Reetam Ganguli, (2023)[17] | USA | Privacy-Preserving | Healthcare Systems | GRNN, LSTM | Real-world metastatic cancer patient cohort | 167,474 patients | Achieving an accuracy of 93.2% with a loss of 0.21%. This synthetic data accurately captured the underlying statistical properties, principal component distributions, and intervariable correlations of the original data, and was shown to be highly effective for training ML classifiers | |
| Zi-Hao Bo, (2023)[18] | China | Physical Isolation, Asynchronous Transfer | Medical Imaging Systems | GAN | Retina Fundus (public) & Hospital CT/MRI Datasets | 41,038 patients | Relay Learning significantly improved model performance across three complex medical imaging tasks compared to sequential and local learning, achieving average performance increases of 44.4% for retinal fundus segmentation, 24.2% for mediastinum tumor diagnosis, and 36.7% for brain midline localization. | |
| Jiaxing Liu, (2023)[19] | Australia | Privacy-Preserving | Electronic Health Records | NLP | OpenDeID pipeline using Discharge Summary BioBERT | 38,414 discharge summaries | Achieved the highest performance in deidentifying sensitive health information (SHI) in Australian pathology reports. Yielded a microaveraged F1-score of 0.9659 for strict matching and an improvement in the strict microaveraged F1-score by 3.04%. | |
| Shalini Gupta, (2024)[20] | India | Privacy Preserving | Electronic Health Records | NLP | OpenDeID corpus | - | Yielded the most favourable performance in automatic EHR deidentification. This specific configuration achieved high micro-averaged F1-scores of 0.9248 for strict matching and 0.9692 for relaxed matching, underscoring the efficacy and potential of the model for protecting patient privacy across varied clinical narratives and geographical regions. | |
| Ruey-Kai Sheu, (2023)[21] | Taiwan | Privacy Preserving | Medical data | SVC | Public Datasets (UCI Heart Disease: Cleveland, Hungary, V.A.) | - | The system demonstrated that FHE can be practically applied in a cloud network, yielding the highest prediction accuracy of 88% by SVC and achieving the best total protocol execution time of 4.23 seconds by the DT algorithm when evaluated across multiple heart disease datasets | |
| T.V. Nguyen, (2022)[22] | Australia | Privacy Preserving | Medical data | FL | Medical (IVF embryo images) & Non-medical (Cat/Dog images) | - | Can exceed the accuracy of centralised training when using real-world noisy data, demonstrating an improvement of up to 11% in accuracy. Furthermore, achieves this high performance while being highly scalable and dramatically reducing data transfer costs (by up to 187.5 times compared to full distributed training), thus protecting data privacy. | |
| Sarvesh Kumar, (2022)[23] | Ghana | Privacy-Protection | Electronic health records | ANN & Fuzzy logic | Personal Health Records (PHR) | - | Achieved an average accuracy rate of 96% across different datasets and reduced the time to find the critical condition from 1,721ms to 72ms. Thereby improving the efficiency of classifying patient criticality. | |
| Uliyan Almousa, (2023)[24] | Saudi Arabia | Anti-Spoofing | Medical Employee Email | Multinomial NB | Kaggle Phishing Email Collection | 525,754 emails | MNB achieved a high F1-score of 96.66% for URL-based bogus website detection and 91.41% for hyperlink-based detection, confirming its reliability across different feature sets. | |
| Gayathri S, (2023)[25] | India | Privacy-Preserving | Medical Image Systems | DNN | Real medical images | - | Effectively preserves medical image privacy in the cloud through secure denoising using deep learning. Achieved high performance metrics, yielding an approximate Signal to Noise Ratio (SNR) of 24.3165 and a Structural Similarity index (SI) of 0.973, while maintaining a low Error Rate (ER) of 0.001 for processed medical images. | |
| Xiaoyuan Gao, (2025)[26] | China | Privacy-Preserving | Medical data | GAN | Public benchmark datasets, Kaggle Cardiovascular disease | 48,842 samples | Excels in balancing privacy protection and data utility across different datasets for intelligent healthcare applications. Specifically, the DP-GAN-HD model achieved high accuracy (up to 0.823 on the KCD dataset with a privacy budget of 2.0) that significantly outperformed other differential privacy models while demonstrating enhanced resistance to privacy attacks. | |
| Bayan Altalla, (2025)[27] | Jordan | Privacy-Preserving | Medical data | LLM GPT 4 | Real EHR | 100 discharge summaries | De-identifying Protected Health Information (PHI) from clinical notes across all evaluated metrics. Specifically, GPT-4 demonstrated superior capability by recording a precision of 0.9925, an F1 score of 0.8973, and an exceptional accuracy rate of 0.9911, confirming its robustness for real-world applications requiring strict privacy adherence | |
| Sultan Mesfer Aldossary, (2024)[28] | Saudi Arabia | Privacy-Preserving | Medical imaging systems | GAN | Open-source medical data | 138 patients | Successfully provides a reliable key generation method to encrypt and decode medical pictures with high security, which effectively preserves the confidentiality and privacy of medical images while making them completely distinct from the originals. | |
| Mahmuda Akter, (2024)[29] | Australia | Privacy-Preserving | Healthcare Systems | FL, CNN | MNIST | 70,000 samples | Successfully integrates secure client adaptation (insertion and removal) and intruder detection without compromising system accuracy or privacy. Specifically, the proposed framework reported a high comparative accuracy of approximately 90% on the MNIST dataset, demonstrating superior performance compared to peer methods which reported 81% | |
| Jitendra Jonnagaddala, (2021)[30] | Australia | Privacy-Preserving | Healthcare Systems | NLP | OpenDeID corpus | 4548 patients | While pre-annotations significantly reduced the total time spent (27.75 hours in Setting 3 vs. 37.4 hours in Setting 1), this approach also resulted in a statistically significant decrease in annotation quality (Inter Annotator Agreement) compared to serial annotations. | |

| **Table 3. Studies Addressing the ‘Detect’ Function of the NIST Cybersecurity Framework (NIST-CSF 2.0)** | | | | | | | |
| --- | --- | --- | --- | --- | --- | --- | --- |
| **Lead Author** | **Country of Origin** | **Aim of Cyber Resilience** | **Healthcare settings** | **AI Model Methodology** | **Training Data & Source** | **Sample Size** | **Key Findings and Outcomes** |
| Akshay Kumaar, (2022)[31] | India | Intrusion Detection | Healthcare Networks | Neural Networks (ImmuneNet) | CIC IDS 2017, IDS 2018, Bell DNS 2021 | - | ImmuneNet achieved the highest accuracy values for all tested datasets, including 99.78% accuracy on the CIC IDS 2018 dataset and 99.19% accuracy on the CIC Bell DNS 2021 dataset, while its lightweight architecture (containing less than 1 million parameters) makes it suitable for deployment on medical devices and healthcare systems |
| Tabassum, (2024)[32] | UK | Anomaly-based threat detection | Electronic Health Records | SVM, DT, Random Forest | EHR audit logs from UK hospital | 1,007,727 entries | Yielded an accuracy of 99.21% and was uniquely capable of identifying new contextual anomalies, such as unauthorized access occurring for abnormal durations after a patient's discharge date, that were missed by the baseline approach. |
| William Hurst, (2022)[33] | UK | Insider-threat detection, anomaly detection | Electronic Health Records | SVM, DT, RF | Real EHR dataset | - | The SVM demonstrated superior performance, achieving a high accuracy of 0.9896 on the test set and 0.9908 on the validation set, leading to the recommendation of its adoption for internal-facing anomaly detection systems |
| N Saranya, (2024)[34] | India | Intrusion Detection | Healthcare Networks | SVM | Public datasets (CICIDS-2017) | - | Experimental results showed a high true positive rate of 96.5% and a low false-positive rate of 2.3%, demonstrating the system's scalability and resilience in successfully protecting network settings |
| Tolgahan Öztürk, (2022)[35] | Turkey | Intrusion Detection | Healthcare Systems | DT | Supervisory Control and Data Acquisition (SCADA) systems in healthcare | 41,403 attack events | Achieved the highest accuracy ratio of 98.8809% and the fastest prediction time of 1240.7 nanoseconds per intrusion. This superior performance holds true when considering both classification accuracy (outperforming SVM and KNN) and the critical factor of rapid detection time for SCADA systems. |
| Mohammad Wazid, (2024)[36] | India | Intrusion detection | Healthcare Systems | SVM, KNN, DT | NSL-KDD dataset | 160,340 samples | Achieved the best accuracy value of approximately 95.12% when utilizing the SKD ensemble method. This proposed system outperformed existing intrusion detection schemes in terms of accuracy while maintaining a comparable linear computational complexity. |
| Sudhakar Sengan, (2022)[37] | India | Intrusion Detection | Healthcare systems | HMM | Real EHR | 63,050 patient data | Effectively detects Denial of Service (DoS) attacks on healthcare data with high precision, achieving an attack detection accuracy of over 98.19%. Furthermore, the results indicate that DAR-ML significantly increases the costs of starting DoS attacks and avoids them by using inter-domain route path identifiers |
| Phong Thanh Nguyen, (2021)[38] | Vietnam | Intrusion Detection | Healthcare network | CNN, LSTM | NSLKDDCup99 Dataset | 148,517 records | Model demonstrated its effectiveness by yielding a maximum accuracy of 99.21%, a precision of 98.93%, and a detection rate of 99.59%. |
| Saad Awadh Alanazi, (2024)[39] | Saudi Arabia | Spear Phishing Attack detection | Healthcare Systems | CNN | Phishing Email Detection dataset from Kaggle | 18,600 email entries | Achieved the highest accuracy at 99.99%, closely followed by the Bidirectional Long Short-Term Memory (BiLSTM) model at 99.94%. This highlights the superior performance of advanced sequential and hierarchical models in effectively detecting SPAs compared to traditional machine learning approaches. |
| Sarah Shihab Ahmed, (2024)[40] | Iraq | Intrusion detection | Healthcare Systems | KNN, RF and DT | Multiple Kaggle Database Dataset | - | Random Forest (RF) algorithm achieving exceptional performance across all types of assaults. Furthermore, the deployment of the MLCAH (Machine Learning-based Cyber Attacks Healthcare) framework demonstrated significant enhancement of network KPIs, leading to a substantial 574% increase in overall performance and a reduction in latency and jitter |
| Laila M. Halman, (2023)[41] | 2023 | Cyberattack detection, impact mitigation | Healthcare Systems | KNN, DT, RF, NB, XGB | Generated dataset of normal and attack traffic | 450,626 samples | Demonstrated impressive reliability with an F1-score of 0.9998 for normal traffic and 0.9882 for attack classes. Significantly enhanced network KPIs by increasing throughput by 609% and reducing delay by 77% and jitter by 23% compared to network performance under attack |
| Muhammad Waseem Asif, (2025)[42] | Pakistan | Intrusion detection and Mitigation | Healthcare Systems | SVM, KNN | NSL-KDD dataset | - | Hybrid SVM- KNN model demonstrated superior performance in Software-Defined Networking (SDN) environments, achieving an overall 30% advancement in detection and mitigation compared to traditional methods like Fuzzy, Logistic Regression, and Decision Tree. |
| Sibo Qiao, (2025)[43] | China | Intrusion detection and prevention | Healthcare networks | Neural networks | - | - | Exhibits superior robustness and efficiency in detecting privacy data leakage in digital healthcare systems. Achieved a Watermark Recognition Accuracy (WRA) above 90% even under high network perturbations outperforming other classical Network Flow Watermarking (NFW) methods |

| **Table 4. Studies Addressing the ‘Respond’ Function of the NIST Cybersecurity Framework (NIST-CSF 2.0)** | | | | | | | |
| --- | --- | --- | --- | --- | --- | --- | --- |
| **Lead Author** | **Country of Origin** | **Aim of Cyber Resilience** | **Healthcare settings** | **AI Model Methodology** | **Training Data & Source** | **Sample Size** | **Key Findings and Outcomes** |
| Lorenzo Fernández Maimó, (2019)[44] | Spain | Ransomware spread detection and mitigation | Cyber-Physical Systems | SVM, NB | Public datasets (ransomware datasets for ICE) | 150,537 samples | The system achieved high detection performance with OC-SVM (99.97% recall) and Naive Bayes classification (99.99% accuracy), and crucially, was able to detect and mitigate the ransomware attack in under 30 seconds, which is fast enough to stop the spread before the fastest ransomware tested could infect a second device (63.1 s). |
| Mustufa Haider Abidi, (2023)[45] | Saudi Arabia | Threat Detection and Blocking | Healthcare Systems | CMPL | University of Queensland Vital Signs (UQVS) dataset | 16,000 samples | Achieved the highest performance in detecting adversarial attacks on patient medical records in smart healthcare systems. Specifically, the CMLP model demonstrated superior results compared to existing approaches, attaining a maximum accuracy of 97%, a precision of 93%, a recall of 92%, and an F1-score of 92% |

| **Table 5. AI/ML Techniques Used in Reviewed Studies (N=45)** | | | |
| --- | --- | --- | --- |
| **Category** | **Technique** | **n** | **Percentage (%)** |
| **Classical Machine Learning** |  |  |  |
|  | SVM | 9 | 20.0 |
|  | DT | 7 | 15.6 |
|  | KNN | 6 | 13.3 |
|  | RF | 6 | 13.3 |
|  | NB | 3 | 6.7 |
|  | BN | 1 | 2.2 |
|  | XGB | 1 | 2.2 |
| **Deep Learning** |  |  |  |
|  | CNN | 6 | 13.3 |
|  | DNN | 5 | 11.1 |
|  | LSTM | 4 | 8.9 |
|  | ANN | 4 | 8.9 |
|  | GAN | 4 | 8.9 |
|  | RNN | 1 | 2.2 |
|  | GRU | 1 | 2.2 |
|  | GRNN | 1 | 2.2 |
|  | DRNN | 1 | 2.2 |
| **Natural Language Processing** |  |  |  |
|  | NLP | 6 | 13.3 |
|  | LLM GPT-4 | 1 | 2.2 |
| **Privacy-Preserving ML** |  |  |  |
|  | FL | 5 | 11.1 |
| **Other Techniques** |  |  |  |
|  | Fuzzy Logic | 3 | 6.7 |
|  | HMM | 1 | 2.2 |
|  | ANFIS | 1 | 2.2 |
|  | ImmuneNet | 1 | 2.2 |
|  | CMPL | 1 | 2.2 |

**Note:** Percentages calculated from total studies (N=45). Some studies employed multiple AI/ML techniques; therefore, totals may exceed 100%.

| **Table 6: Abbreviations for Machine Learning Terminologies** | |
| --- | --- |
| **Abbreviation** | **Full Form** |
| AI | Artificial Intelligence |
| ANN | Artificial Neural Network |
| ANFIS | Adaptive Neuro-Fuzzy Inference System |
| BN | Bayesian Network |
| CMLP | Crossover-based Multilayer Perceptron |
| CNN | Convolutional Neural Network |
| DNN | Deep Neural Network |
| DRNN | Deep Recurrent Neural Network |
| DT | Decision Tree |
| FL | Federated Learning |
| Fuzzy logic | Fuzzy Logic System |
| GAN | Generative Adversarial Network |
| GPT-4 | Generative Pretrained Transformer version 4 |
| GRNN | Generalized Regression Neural Network |
| GRU | Gated Recurrent Unit |
| HMM | Hidden Markov Model |
| KNN | K-Nearest Neighbours / K-Nearest Neighbors |
| LLM | Large Language Model |
| LSTM | Long Short-Term Memory |
| NB | Naive Bayes |
| NLP | Natural Language Processing |
| RF | Random Forest |
| RNN | Recurrent Neural Network |
| SVC | Support Vector Classifier |
| SVM | Support Vector Machine |
| XGB | XGBoost (Extreme Gradient Boosting) |

## References

1. Unozkan H, Ertem M, Bendak S. Using attack graphs to defend healthcare systems from cyberattacks: a longitudinal empirical study. Netw Model Anal Health Inform Bioinform. 2022;11(1):52. PMID: 36408329. doi: 10.1007/s13721-022-00391-1.

2. Kaur J, Khan AI, Abushark YB, Alam MM, Khan SA, Agrawal A, et al. Security Risk Assessment of Healthcare Web Application Through Adaptive Neuro-Fuzzy Inference System: A Design Perspective. Risk Manag Healthc Policy. 2020;13:355–71. PMID: 32425625. doi: 10.2147/RMHP.S233706.

3. Silvestri S, Islam S, Papastergiou S, Tzagkarakis C, Ciampi M. A Machine Learning Approach for the NLP-Based Analysis of Cyber Threats and Vulnerabilities of the Healthcare Ecosystem. Sensors (Basel). 2023 Jan 6;23(2). PMID: 36679446. doi: 10.3390/s23020651.

4. Li G, Dong Z, Wang Y. Information security of hospital computer network based on SAE deep neural network. Applied Mathematics and Nonlinear Sciences. 2024;9(1). doi: 10.2478/amns.2023.1.00466.

5. Islam S, Abba A, Ismail U, Mouratidis H, Papastergiou S. Vulnerability prediction for secure healthcare supply chain service delivery. Integrated Computer-Aided Engineering. 2022;29(4):389–409. doi: 10.3233/ica-220689.

6. Yi X, Wu J, Li G, Bashir AK, Li J, AlZubi AA. Recurrent Semantic Learning-Driven Fast Binary Vulnerability Detection in Healthcare Cyber Physical Systems. IEEE Transactions on Network Science and Engineering. 2023;10(5):2537–50. doi: 10.1109/tnse.2022.3199990.

7. Hafiz Mohd Yusof M, Mohd Zin A, Safie Mohd Satar N. Behavioral Intrusion Prediction Model on Bayesian Network over Healthcare Infrastructure. Computers, Materials & Continua. 2022;72(2):2445–66. doi: 10.32604/cmc.2022.023571.

8. Dolezel D, Beauvais B, Stigler Granados P, Fulton L, Kruse CS. Effects of Internal and External Factors on Hospital Data Breaches: Quantitative Study. J Med Internet Res. 2023 Dec 21;25:e51471. PMID: 38127426. doi: 10.2196/51471.

9. Gwon H, Ahn I, Kim Y, Kang HJ, Seo H, Choi H, et al. LDP-GAN : Generative adversarial networks with local differential privacy for patient medical records synthesis. Comput Biol Med. 2024 Jan;168:107738. PMID: 37995536. doi: 10.1016/j.compbiomed.2023.107738.

10. Goldschmidt G, Zeiser FA, Righi RDR, Da Costa CA. ARTERIAL: A Natural Language Processing Model for Prevention of Information Leakage from Electronic Health Records. 2023 XIII Brazilian Symposium on Computing Systems Engineering (SBESC)2023. p. 1–6.

11. Huang T, Xu J, Tu S, Han B. Robust zero-watermarking scheme based on a depthwise overparameterized VGG network in healthcare information security. Biomedical Signal Processing and Control. 2023;81. doi: 10.1016/j.bspc.2022.104478.

12. Akter M, Moustafa N, Lynar T, Razzak I. Edge Intelligence: Federated Learning-Based Privacy Protection Framework for Smart Healthcare Systems. IEEE J Biomed Health Inform. 2022 Dec;26(12):5805–16. PMID: 35857737. doi: 10.1109/JBHI.2022.3192648.

13. An Z, Zhang J, Jiang Z, Du J, Yin Z, Li C. FedMCC: Federated multi-center clustering algorithm to improve privacy healthcare. Methods. 2023 Oct;218:94–100. PMID: 37507060. doi: 10.1016/j.ymeth.2023.07.006.

14. Nasibeh Mohammadi AR, Seyd Hamid Haj Seydjavadi, Parvaneh Asghari. FLHB-AC: Federated Learning History-Based Access Control Using Deep

Neural Networks in Healthcare System. Journal of Information Systems and Telecommunication. 2024;12(2).

15. Alzubi JA, Alzubi OA, Beseiso M, Budati AK, Shankar K. Optimal multiple key‐based homomorphic encryption with deep neural networks to secure medical data transmission and diagnosis. Expert Systems. 2021;39(4). doi: 10.1111/exsy.12879.

16. Cabrero-Holgueras J, Pastrana S. Towards realistic privacy-preserving deep learning over encrypted medical data. Front Cardiovasc Med. 2023;10:1117360. PMID: 37187785. doi: 10.3389/fcvm.2023.1117360.

17. Ganguli R, Lad R, Lin A, Yu X. Novel Generative Recurrent Neural Network Framework to Produce Accurate, Applicable, and Deidentified Synthetic Medical Data for Patients With Metastatic Cancer. JCO Clin Cancer Inform. 2023 May;7:e2200125. PMID: 37130342. doi: 10.1200/CCI.22.00125.

18. Bo ZH, Guo Y, Lyu J, Liang H, He J, Deng S, et al. Relay learning: a physically secure framework for clinical multi-site deep learning. NPJ Digit Med. 2023 Nov 4;6(1):204. PMID: 37925578. doi: 10.1038/s41746-023-00934-4.

19. Liu J, Gupta S, Chen A, Wang CK, Mishra P, Dai HJ, et al. OpenDeID Pipeline for Unstructured Electronic Health Record Text Notes Based on Rules and Transformers: Deidentification Algorithm Development and Validation Study. J Med Internet Res. 2023 Dec 6;25:e48145. PMID: 38055317. doi: 10.2196/48145.

20. Gupta S, Liu J, Wong ZS, Jonnagaddala J. Preliminary Evaluation of Fine-Tuning the OpenDeLD Deidentification Pipeline Across Multi-Center Corpora. Stud Health Technol Inform. 2024 Aug 22;316:719–23. PMID: 39176896. doi: 10.3233/SHTI240515.

21. Sheu RK, Lin YC, Pardeshi MS, Huang CY, Pai KC, Chen LC, et al. Adaptive Autonomous Protocol for Secured Remote Healthcare Using Fully Homomorphic Encryption (AutoPro-RHC). Sensors (Basel). 2023 Oct 16;23(20). PMID: 37896596. doi: 10.3390/s23208504.

22. Nguyen TV, Dakka MA, Diakiw SM, VerMilyea MD, Perugini M, Hall JMM, et al. A novel decentralized federated learning approach to train on globally distributed, poor quality, and protected private medical data. Sci Rep. 2022 May 25;12(1):8888. PMID: 35614106. doi: 10.1038/s41598-022-12833-x.

23. Kumar S, Wajeed MA, Kunabeva R, Dwivedi N, Singhal P, Jamal SS, et al. Novel Method for Safeguarding Personal Health Record in Cloud Connection Using Deep Learning Models. Comput Intell Neurosci. 2022;2022:3564436. PMID: 35345805. doi: 10.1155/2022/3564436.

24. Bander Nasser Almousa DMU. Anti-Spoofing in Medical Employee's Email using

Machine Learning Uclassify Algorithm. International Journal of Advanced Computer Science and Applications. 2023;14(7).

25. S G, S G. Securing medical image privacy in cloud using deep learning network. Journal of Cloud Computing. 2023;12(1). doi: 10.1186/s13677-023-00422-w.

26. Gao X, Mi W, Feng X. Personal health data protection and intelligent healthcare applications under generative adversarial network. Sci Rep. 2025 May 13;15(1):16558. PMID: 40360631. doi: 10.1038/s41598-025-01575-1.

27. Altalla B, Abdalla S, Altamimi A, Bitar L, Al Omari A, Kardan R, et al. Evaluating GPT models for clinical note de-identification. Sci Rep. 2025 Jan 31;15(1):3852. PMID: 39890969. doi: 10.1038/s41598-025-86890-3.

28. Mesfer Aldossary S. DeepGan-Privacy Preserving of HealthCare System Using DL. Intelligent Automation & Soft Computing. 2023;37(2):2199–212. doi: 10.32604/iasc.2023.038243.

29. Akter M, Moustafa N, Turnbull B. SPEI-FL: Serverless Privacy Edge Intelligence-Enabled Federated Learning in Smart Healthcare Systems. Cognitive Computation. 2024;16(5):2626–41. doi: 10.1007/s12559-024-10310-3.

30. Jonnagaddala J, Chen A, Batongbacal S, Nekkantti C. The OpenDeID corpus for patient de-identification. Scientific Reports. 2021;11(1). doi: 10.1038/s41598-021-99554-9.

31. Akshay Kumaar M, Samiayya D, Vincent P, Srinivasan K, Chang CY, Ganesh H. A Hybrid Framework for Intrusion Detection in Healthcare Systems Using Deep Learning. Front Public Health. 2021;9:824898. PMID: 35096763. doi: 10.3389/fpubh.2021.824898.

32. Tabassum M, Mahmood S, Bukhari A, Alshemaimri B, Daud A, Khalique F. Anomaly-based threat detection in smart health using machine learning. BMC Med Inform Decis Mak. 2024 Nov 19;24(1):347. PMID: 39563355. doi: 10.1186/s12911-024-02760-4.

33. Hurst W, Tekinerdogan B, Alskaif T, Boddy A, Shone N. Securing electronic health records against insider-threats: A supervised machine learning approach. Smart Health. 2022;26. doi: 10.1016/j.smhl.2022.100354.

34. Saranya N, Abbinavu T, Kumar PA, Gnanasekar R, Prasanth PG, Subha R. RTIDS: A Robust Transformer-based Approach for Intrusion Detection System. 2024 10th International Conference on Advanced Computing and Communication Systems (ICACCS)2024. p. 1461–4.

35. Öztürk T, Turgut Z, Akgün G, Köse C. Machine learning-based intrusion detection for SCADA systems in healthcare. Network Modeling Analysis in Health Informatics and Bioinformatics. 2022;11(1). doi: 10.1007/s13721-022-00390-2.

36. Wazid M, Singh J, Das AK, Rodrigues JJPC. An Ensemble-Based Machine Learning-Envisioned Intrusion Detection in Industry 5.0-Driven Healthcare Applications. IEEE Transactions on Consumer Electronics. 2024;70(1):1903–12. doi: 10.1109/tce.2023.3318850.

37. Sengan S, Khalaf OI, Vidya Sagar P, Sharma DK, Arokia Jesu Prabhu L, Hamad AA. Secured and Privacy-Based IDS for Healthcare Systems on E-Medical Data Using Machine Learning Approach. International Journal of Reliable and Quality E-Healthcare. 2021;11(3):1–11. doi: 10.4018/ijrqeh.289175.

38. Thanh Nguyen P, Dang Bich Huynh V, Dang Vo K, Thanh Phan P, Elhoseny M, Le D-N. Deep Learning based Optimal Multimodal Fusion Framework for Intrusion Detection Systems for Healthcare Data. Computers, Materials & Continua. 2021;66(3):2555–71. doi: 10.32604/cmc.2021.012941.

39. Alanazi SA. ML-SPAs: Fortifying Healthcare Cybersecurity Leveraging Varied Machine Learning Approaches against Spear Phishing Attacks. Computers, Materials & Continua. 2024;81(3):4049–80. doi: 10.32604/cmc.2024.057211.

40. Sarah Shihab Ahmed HRS. Enhancement of Secure Hospital Healthcare Monitoring System

Based–Software Defined Network (SDN) with Machine Learning. INTERNATIONAL JOURNAL

ON INFORMATICS VISUALIZATION. 2024;8(4).

41. Halman LM, Alenazi MJF. MCAD: A Machine Learning Based Cyberattacks Detector in Software-Defined Networking (SDN) for Healthcare Systems. IEEE Access. 2023;11:37052–67. doi: 10.1109/access.2023.3266826.

42. Asif MW, Aqdus A, Amin R, Chaudhry SA, Alsubaei FS, Iqbal S. An Efficient Intrusion Detection System using Advanced Machine Learning Techniques in Software-Defined Networks (SDN) for Healthcare System. IEEE J Biomed Health Inform. 2025 Jan 16;PP. PMID: 40030900. doi: 10.1109/JBHI.2025.3530563.

43. Qiao S, Guo Q, Shi F, Wang M, Zhu H, Khan F, et al. SIBW: A Swarm Intelligence-Based Network Flow Watermarking Approach for Privacy Leakage Detection in Digital Healthcare Systems. IEEE J Biomed Health Inform. 2025 Feb 14;PP. PMID: 40036416. doi: 10.1109/JBHI.2025.3542561.

44. Fernandez Maimo L, Huertas Celdran A, Perales Gomez AL, Garcia Clemente FJ, Weimer J, Lee I. Intelligent and Dynamic Ransomware Spread Detection and Mitigation in Integrated Clinical Environments. Sensors (Basel). 2019 Mar 5;19(5). PMID: 30841592. doi: 10.3390/s19051114.

45. Abidi MH, Alkhalefah H, Aboudaif MK. Enhancing Healthcare Data Security and Disease Detection Using Crossover-Based Multilayer Perceptron in Smart Healthcare Systems. Computer Modeling in Engineering & Sciences. 2024;139(1):977–97. doi: 10.32604/cmes.2023.044169.
