# Supplementary material for: Mapping Machine Learning–Driven Cybersecurity Solutions in Health Care: Scoping Literature Review
Source: J Med Internet Res. 2026 Jul 27;28:e93950. doi: 10.2196/93950 (PMC13405368; doi:10.2196/93950)
Supplement: Checklist 2 [file jmir-v28-e93950-s004.docx]

**PRISMA-S Checklist**

| **Section/topic** | **#** | **Checklist item** | **Location(s) Reported** |
| --- | --- | --- | --- |
| **INFORMATION SOURCES AND METHODS** | | | |
| Database name | 1 | Name each individual database searched, stating the platform for each. | Page 6  Methods: Ovid MEDLINE (Ovid platform); Embase Classic+Embase (Ovid platform); Scopus (native interface) |
| Multi-database searching | 2 | If databases were searched simultaneously on a single platform, state the name of the platform, listing all of the databases searched. | Page 6  Methods: MEDLINE and Embase Classic+Embase were searched simultaneously via the Ovid platform; Scopus was searched separately via its native interface |
| Study registries | 3 | List any study registries searched. | Page 7  Not applicable: no study registries were searched. This review was restricted to peer-reviewed empirical studies indexed in bibliographic databases. |
| Online resources and browsing | 4 | Describe any online or print source purposefully searched or browsed (e.g., tables of contents, print conference proceedings, web sites), and how this was done. | Page 7  Not applicable: no websites, tables of contents, or print sources were purposefully browsed. This review was restricted to peer-reviewed empirical studies indexed in the three bibliographic databases; this is acknowledged as a limitation. |
| Citation searching | 5 | Indicate whether cited references or citing references were examined, and describe any methods used for locating cited/citing references (e.g., browsing reference lists, using a citation index, setting up email alerts for references citing included studies). | Page 9  Results: included articles were manually screened to identify any additional eligible studies |
| Contacts | 6 | Indicate whether additional studies or data were sought by contacting authors, experts, manufacturers, or others. | Page 7  Not applicable: no authors, experts, or manufacturers were contacted to identify additional studies. |
| Other methods | 7 | Describe any additional information sources or search methods used. | Page 6  Not applicable: no additional information sources or search methods beyond database searching and reference list screening were used. |
| **SEARCH STRATEGIES** | | | |
| Full search strategies | 8 | Include the search strategies for each database and information source, copied and pasted exactly as run. | Page 6  Multimedia Appendix 2: full search strategies for Ovid MEDLINE, Embase Classic+Embase, and Scopus copied exactly as run, including all limits |
| Limits and restrictions | 9 | Specify that no limits were used, or describe any limits or restrictions applied to a search (e.g., date or time period, language, study design) and provide justification for their use. | Page 7  Methods: searches limited to English language, publication years 2019–2025, and human studies where database functionality permitted. Restricted to 2019–2025 to capture recent developments in AI applications for cybersecurity in healthcare. |
| Search filters | 10 | Indicate whether published search filters were used (as originally designed or modified), and if so, cite the filter(s) used. | Page 7  Methods: no published search filters were used. Database-native limits for language, date, and publication type were applied directly. |
| Prior work | 11 | Indicate when search strategies from other literature reviews were adapted or reused for a substantive part or all of the search, citing the previous review(s). | Page 7  Methods: the search strategy was developed de novo in consultation with an expert librarian at Imperial College London and was not adapted or reused from any prior review. |
| Updates | 12 | Report the methods used to update the search(es) (e.g., rerunning searches, email alerts). | Page 7  The search was originally conducted on 30 July 2025. Following reviewer and editor feedback, the search strategy was revised and rerun during the revision process; however, the search end date of 30 July 2025 was retained as the fixed time horizon for the review. No email alerts were employed. |
| Dates of searches | 13 | For each search strategy, provide the date when the last search occurred. | Page 7  The original search was conducted on 30 July 2025 across all three databases. Following reviewer and editor feedback, the revised search strategy was rerun across all three databases during the revision process, with 30 July 2025 retained as the fixed end date. Both the original and revised search strategies are available in Multimedia Appendix 2. |
| **PEER REVIEW** | | | |
| Peer review | 14 | Describe any search peer review process. | Page 7  Methods: The search strategy was developed in consultation with an expert librarian at Imperial College London. Independent peer review process was not conducted. |
| **MANAGING RECORDS** | | | |
| Total Records | 15 | Document the total number of records identified from each database and other information sources. | Page 8  Results (PRISMA-ScR flow diagram, Figure 1) and Methods: Embase n=2,521; MEDLINE n=4,527; Scopus n=33,00; total n=10,348 prior to deduplication |
| Deduplication | 16 | Describe the processes and any software used to deduplicate records from multiple database searches and other information sources. | Page 8  Results: deduplication was performed using the Covidence platform (Veritas Health Innovation, Melbourne, Australia); 2,014 duplicate records were identified and removed |
|  |  |  |  |
| PRISMA-S: An Extension to the PRISMA Statement for Reporting Literature Searches in Systematic Reviews | | |  |
| Rethlefsen ML, Kirtley S, Waffenschmidt S, Ayala AP, Moher D, Page MJ, Koffel JB, PRISMA-S Group. | | |  |
| Last updated February 27, 2020. | |  |  |
